# Self-expanding metal stents versus decompression tubes as a bridge to surgery for patients with obstruction caused by colorectal cancer: a systematic review and meta-analysis

**DOI:** 10.1186/s13017-023-00515-6

**Published:** 2023-09-27

**Authors:** Wei Ma, Jian-Cheng Zhang, Kun Luo, Lu Wang, Chi Zhang, Bin Cai, Hua Jiang

**Affiliations:** 1 School of Medicine and Life Sciences, Chengdu University of Traditional Chinese Medicine, Chengdu, 611137 China; 2 Institute for Emergency and Disaster Medicine, Sichuan Academy of Medical Sciences, Sichuan Provincial People’s Hospital, School of Medicine, University of Electronic Science and Technology of China, Sichuan Province, No. 32, Yi Huan Lu Xi Er Duan, Chengdu, 610072 China; 3 Sichuan Provincial Center for Emergency Medicine, Sichuan Academy of Medical Sciences, Sichuan Provincial People’s Hospital, School of Medicine, University of Electronic Science and Technology of China, Chengdu, 610072 China; 4 Sichuan Provincial Research Center for Emergency Medicine and Critical Illness. Sichuan Academy of Medical Sciences, Sichuan Provincial People’s Hospital, School of Medicine, University of Electronic Science and Technology of China, Chengdu, 610072 China

**Keywords:** Colorectal cancer, Bowel obstruction, Self-expanding metal stents, Decompression tubes, Bridge to surgery

## Abstract

**Background:**

Using self-expanding metal stents (SEMS) and decompression tubes (DT) as a bridge-to-surgery (BTS) treatment may avoid emergency operations for patients with colorectal cancer-caused obstructions. This study aimed to evaluate the efficacy and safety of the two approaches.

**Methods:**

We systematically retrieved literature from January 1, 2000, to May 30, 2023, from the PubMed, Embase, Web of Science, SinoMed, Wanfang Data, Chinese National Knowledge Infrastructure, and Cochrane Central Register of Clinical Trials databases. Randomized controlled trials (RCTs) or cohort studies of SEMS versus DT as BTS in colorectal cancer obstruction were selected. Risks of bias were assessed for RCTs and cohort studies using the Cochrane Risk of Bias tool version 2 and Risk of Bias in Nonrandomized Studies of Interventions. Certainty of evidence was determined using the Graded Recommendation Assessment. Odds ratio (OR), mean difference (MD), and 95% confidence interval (95% CI) were used to analyze measurement data.

**Results:**

We included eight RCTs and eighteen cohort studies involving 2,061 patients (SEMS, 1,044; DT, 1,017). Pooled RCT and cohort data indicated the SEMS group had a significantly higher clinical success rate than the DT group (OR = 1.99, 95% CI 1.04, 3.81, *P* = 0.04), but no significant difference regarding technical success (OR = 1.29, 95% CI 0.56, 2.96, *P* = 0.55). SEMS had a shorter postoperative length of hospital stays (MD = − 4.47, 95% CI − 6.26, − 2.69, *P* < 0.00001), a lower rates of operation-related abdominal pain (OR = 0.16, 95% CI 0.05, 0.50, *P* = 0.002), intraoperative bleeding (MD = − 37.67, 95% CI − 62.73, − 12.60, *P* = 0.003), stoma creation (OR = 0.41, 95% CI 0.23, 0.73, *P* = 0.002) and long-term tumor recurrence rate than DT (OR = 0.47, 95% CI 0.22, 0.99, *P* = 0.05).

**Conclusion:**

SEMS and DT are both safe as BTS to avoid emergency surgery for patients with colorectal cancer obstruction. SEMS is preferable because of higher clinical success rates, lower rates of operation-related abdominal pain, intraoperative bleeding, stoma creation, and long-term tumor recurrence, as well as a shorter postoperative length of hospital stays.

*Trial registration* CRD42022365951.

**Supplementary Information:**

The online version contains supplementary material available at 10.1186/s13017-023-00515-6.

## Background

According to the latest global cancer burden data released by the International Agency for Research on Cancer of the World Health Organization in 2020, incidence of colorectal cancer ranks third in terms of incidence among all cancers, accounting for approximately 10% of new cancer cases globally. Moreover, it has escalated to the second leading cause of cancer-related deaths worldwide, accounting for approximately 9.4% of all cancer-related deaths [1]. Obstruction is one of the most common complications of colorectal cancer, with a prevalence as high as 29%. It also constitutes a significant percentage of emergency department admissions, as this critical condition often requires emergency interventions [2]. Recent studies have shown an alarmingly high postoperative mortality rate among patients with obstruction caused by colorectal cancer, with a 30-day mortality rate exceeding 50% [3, 4]. Additionally, the risk of perioperative morbidity is increased by the generally poor systemic condition of patients, e.g., electrolyte and acid base imbalances, intestinal congestion and edema [5]. Consequently, any surgical approach to treating these patients may significantly increase the risk of mortality, as well as escalate hospitalization costs and prolong the recovery [6].

In recent years, the endoscopic placement of self-expanding metal stents (SEMS) and decompression tubes (DT) has emerged as a bridge to surgery (BTS), allowing for the rapid relief of obstruction symptoms in patients and avoiding emergency surgery. This approach creates conditions for radical resection, thus improving the overall survival rate of patients. In 1991, Dohmoto et al. [7] first reported the use of endoscopically placed SEMS as a palliative treatment for rectal and sigmoid colon cancers. With the advancement of endoscopic techniques, SEMS can also be used as a transitional tool before radical colorectal cancer resection. Several studies have reported the role of SEMS in relieving obstruction due to colorectal cancer [8–11]. SEMS not only reduces the stoma rate and length of postoperative hospital stay but also decreases the mortality rate in patients with colorectal cancer obstruction [12]. The European Society of Gastrointestinal Endoscopy (ESGE) and the American Society for Gastrointestinal Endoscopy recommend SEMS as an option for palliation and relief of malignant bowel obstruction [13, 14]. The ESGE suggests that SEMS can be used as an alternative to emergency surgery for potentially curable colorectal cancer obstruction.

In 1940, Abbott et al. [15] developed DT, which alleviates intestinal obstruction by repeatedly flushing the intestinal lumen upon reaching the site of obstruction. Studies have demonstrated the safety and efficacy of DT [16–19]. Prior to the healthcare policy reforms in Japan, DT was often the preferred treatment modality for malignant colonic obstruction [20].

Both endoscopically placed SEMS and DT have high technical and clinical success rates [21, 22]. However, the differential effects of SEMS and DT in patients with colorectal cancer obstruction remain controversial. This study aimed to evaluate the effectiveness and safety of SEMS and DT as BTS in relieving colorectal cancer obstruction, as well as to compare the short- and long-term outcomes of subsequent radical resection.

## Methods

This systematic evaluation adheres to the guidelines outlined by Preferred Reporting Items for Systematic Reviews and Meta-Analyses (PRISMA), and our research plan has been registered with PROSPERO (CRD42022365951).

### Search strategy

The literature search was conducted independently by two researchers (WM and J-CZ) using the following databases: PubMed, Embase, Web of Science, SinoMed, Wanfang Data, Chinese National Knowledge Infrastructure, and the Cochrane Central Register of Clinical Trials. The search covered the period from January 1, 2000, to May 1, 2023. The inclusion criteria for the literature were studies published in English or Chinese. The search terms were combined using Boolean logic and connected with "AND/OR" and the search strategies of the mentioned databases can be found in Additional file 1: Table S1.

### Inclusion and exclusion criteria

After importing all the retrieved literature into a reference management software, duplicate articles were removed. Subsequently, two independent researchers (WM and J-CZ) reviewed the titles and abstracts of the remaining articles based on the basis of the inclusion and exclusion criteria to identify potentially relevant studies. The inclusion criteria consisted of the following: (1) randomized controlled trials (RCTs) and cohort studies; (2) participants diagnosed with obstruction caused by colorectal cancer confirmed by abdominal computed tomography (CT) or endoscopic biopsy; (3) interventions involving SEMS and DT; and (4) studies providing relevant indicators, including (a) operation-related outcomes such as technical success, clinical success, and operation-related complications; (b) surgery-related outcomes such as intraoperative bleeding, stoma rate, length of hospital stay, and surgery-related complications; and (c) long-term outcomes such as survival, tumor recurrence, and tumor metastasis. Exclusion criteria included the following: (1) case reports, systematic reviews, and meta-analyses; (2) interventions other than SEMS or DT; and (3) studies lacking the reporting of the aforementioned relevant indicators. When the reviewers disagreed regarding the inclusion of an article, its full text was read to discuss its inclusion. If a consensus could not be reached between the two researchers, the final decision was made by a third researcher (HJ) of the review team.

### Outcome definition

Technical success was defined as the achievement of instrument placement. Clinical success was defined as the resolution of obstructive symptoms. Other outcomes were defined in accordance with the respective definitions of each included study.

### Data extraction

Two independent researchers (WM and J-CZ) assessed the eligibility of selected articles and extracted the following information: study characteristics (first author’s name, publication year, country, and study design), patient characteristics (age, sex sample size, clinical stage, tumor location, and device type), operation-related outcomes (technical success, clinical success, and operation-related complications), surgery-related outcomes (intraoperative bleeding, stoma rate, length of hospital stay, and surgery-related complications), and long-term outcomes (survival, tumor recurrence, and tumor metastasis rate). To minimize data entry errors, all data were entered by the two independent researchers and checked by a third researcher (HJ), with any discrepancies resolved through discussion.

### Assessment of risk of bias

Two independent researchers (J-CZ and KL) independently assessed the risk of bias in the included studies. The risk of bias assessment for RCTs was conducted using the Cochrane Risk of Bias tool version 2 (RoB 2), which evaluates six domains of bias: (1) randomization process, (2) deviations from intended interventions, (3) missing outcome data, (4) measurement of outcomes, (5) selection of reported results, and (6) overall bias [23]. Risk Of Bias in Non-randomized Studies—of Interventions (ROBINS-I), a tool for evaluating the risk of bias in cohort studies [24], includes seven domains: (1) bias due to confounding, (2) bias in participant selection, (3) bias in classification of interventions, (4) bias due to deviations from intended interventions, (5) bias due to missing data, (6) bias in measurements of outcomes, and (7) bias in selection of the reported results. Any discrepancies between the two researchers were resolved by a third researcher (HJ).

### Certainty of evidence

The certainty of the evidence was evaluated by two independent researchers (J-CZ and KL) using the Graded Recommendations Assessment, Development, and Evaluation (GRADE) through the GRADE Pro online website tool [25, 26]. We assessed the quality of the evidence and the confidence in the effect estimates based on study design, risk of bias, inconsistency, indirectness, imprecision, and risk of publication bias. For each outcome, the overall quality of the evidence was described as "high," "moderate," "low," or "very low." Any discrepancies between the two researchers were resolved by a third researcher (HJ).

### Statistical analysis

Methods for assessing heterogeneity in included studies comprised visual inspection and statistical tests. When heterogeneity was absent, a fixed effect model was applied to pool data. If the heterogeneity existed, then a random effect model was applied. Visual inspection often employed a forest plot, where an elevated level of homogeneity could be inferred if the confidence intervals (CIs) overlapped and there were no apparent outliers in the point estimates. Statistical tests, such as the Q test and I^2^ statistic, were also utilized. An I^2^ value ≥ 75% indicates high heterogeneity among the included studies, 50% ≤ I^2^ < 75% suggests moderate heterogeneity, and 25% ≤ I^2^ < 50% indicates low heterogeneity [27]. For indicators with > ten articles, a funnel plot was utilized to evaluate publication bias in the included literature.

When the outcome measures were binary variables, the effect size was evaluated using odds ratios (ORs) and their corresponding 95% CIs. For continuous numerical variables, the effect size was assessed using mean differences (MDs) and their corresponding 95% CIs. If the data were reported in formats other than the mean and standard deviation (e.g., in the case of median and range), we applied the method developed by Hozo et al. [28] to transform them. The statistical significance of the pooled effect size was determined using the Z-test. Unless stated otherwise, a P-value < 0.05 was considered statistically significant. Meta-analysis was conducted using the Review Manager 5.4 software.

## Results

**Fig. 1:**
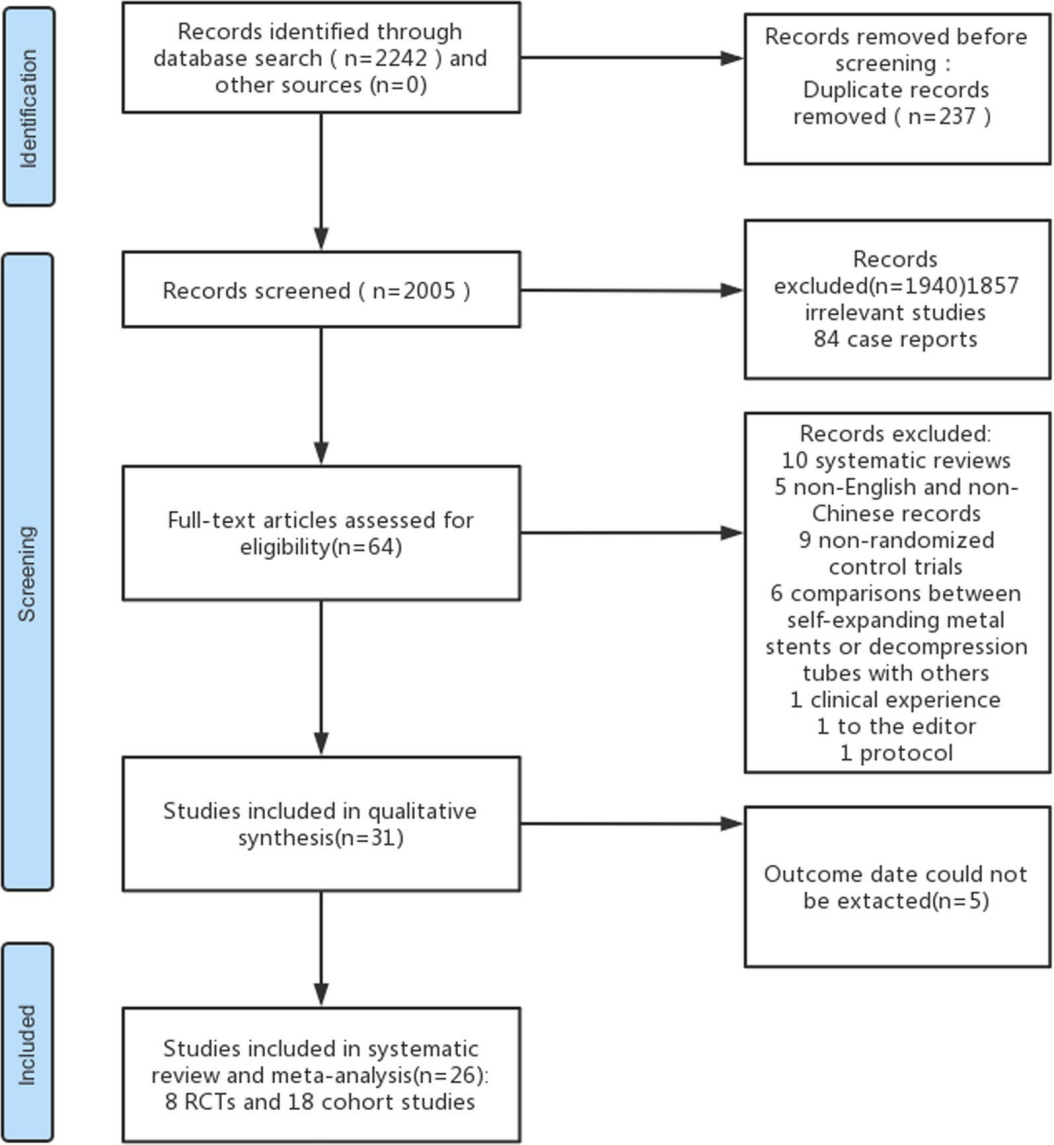
Literature search and selection. RCT, randomized controlled trial

According to the above-mentioned search strategy, a total of 2,242 articles were retrieved from electronic databases spanning January 1, 2000, to May 30, 2023. Detailed insights into the selection process and exclusion rationale are presented through the PRISMA diagram (Fig. 1). Eight RCTs and eighteen cohort studies fulfilled the inclusion criteria, enlisting a cumulative 2,061 participants (Table 1). All eight RCTs originated in China, with seven cohort studies from China and eleven from Japan. Notably, among the studies, eighteen focused on left-sided colon cancer obstructions, while one centered on right-sided colon cancer obstructions, and seven studies covered obstructions in any part of the colon. Sample sizes ranged from 31 to 206 participants, with 1,044 in the SEMS group and 1,017 in the DT group. Participant ages spanned 56.1 to 76.0 years. Diverse SEMS models, encompassing Niti-S, WallFlex, Hanaro, and Naturfit, were adopted, while DT models included Create Medic and Dennis. Further comprehensive trial characteristics are summarized in Additional file 2: Table S2.

**Table 1:** Methodological characteristics of the included studies

Study	Country	Study design	Group	Sample size		Mean age(years)		Sex (male/female)		Tumor location		Stage		Instrument types	
				SEMS	DT	SEMS	DT	SEMS	DT	SEMS	DT	SEMS	DT	SEMS	DT
Yang [6]	China	RCT	SEMS/DT	20	20	60.6	68.8			Descending 4, Sigmoid 6, Rectum 10	Descending 3, Sigmoid 5, Rectum 12			Niti-S, WallFlex	Create Medic
Xin [29]	China	RCT	SEMS/DT	50	50	71 ± 3	71 ± 5	32/18	29/21	Descending 8, Sigmoid l4, Rectum 28	Descending 7, Sigmoid l6, Rectum 27			Niti-S, WallFlex	Create Medic
Chen [30]	China	CS	SEMS/DT	27	18	60.2 ± 2.8	63.8 ± 3.5			Transverse 1, Descending 4, Sigmoid 14, Rectum 8	Descending 3, Sigmoid 8, Rectum 7			Niti-S	Create Medic
Chen [31]	China	RCT	SEMS/DT	40	40	67.00 ± 9.67	66.13 ± 11.11	22/18	24/16	Splenic flexure 6, Descending 10, Sigmoid 13, Rectum 11	Splenic flexure 7, Descending 9, Sigmoid 11, Rectum 13	A 0, B 5, C 20, D 15	A 0, B 8, C 18, D 14	Niti-S	Create Medic
Li [32]	China	CS	SEMS/DT	35	16	68.4 ± 6.9	72.2 ± 8.3	19/16	9/7	Colon 26, Rectum 9	Colon 10, Rectum 6	II 8, III 24, IV 3	II 4, III 10, IV 2		
Akihisa Matsuda [33]	Japan	CS	SEMS/DT	28	45	66 ± 3.25	70 ± 5.25	17/11	29/16	Ascending 5, Transverse 3, Descending 3, Sigmoid 27, Rectum 12	Transverse 3, Descending 3, Sigmoid 27, Rectum 12	II 3, III 17, IV 8	II 4, III 22, IV 19	Niti-S, WallFlex	Dennis
Kojima [34]	Japan	CS	SEMS/DT	27	42	73 ± 17.0	65 ± 15.2	10/17	23/19	Right 4, Left 23	Right 4, Left 38				
Hiroshi Takeyama [35]	Japan	CS	SEMS/DT	22	19	71.3 ± 10.3	68.5 ± 10	8/14	8/4	Transverse 7, Descending 3, Sigmoid 7, Rectum 5	Descending 3, Sigmoid 6, Rectum 3	II 6, III 11, IV 5	II 5, III 5, IV 2	Niti-S, WallFlex	Dennis
Chen [36]	China	CS	SEMS/DT	22	11	72.6 ± 4.7	68.3 ± 5.2	14/8	7/4	Left 22	Left Colon 11			Niti-S	Create Medic
Yang [37]	China	RCT	SEMS/DT	70	70	64.5 ± 9.8	66.5 ± 11.2	45/25	43/27	Hepatic flexure 17, Transverse 4, Descending 7, Sigmoid 27, Rectum 1 5	Hepatic flexure 19, Transverse 4, Descending 7, Sigmoid 27, Rectum 13	A 1, B 24, C 25, D 20	A 0, B 23, C 26, D 21	Niti-S	Create Medic
Chen [38]	China	RCT	SEMS/DT	50	50										
Liu [39]	China	RCT	SEMS/DT	30	30	58.9 ± 10.3	61.2 ± 12.2	19/11	18/12	Transverse 5, Descending 6, Sigmoid 7, Rectum 12	Transverse 6, Descending 7, Sigmoid 6, Rectum 11				
Satoru Kagami [40]	Japan	CS	SEMS/DT	26	33	70 ± 8.75	68 ± 11.0	17/9	23/10	Transverse 2, Descending 3, Sigmoid 17, Rectum 4	Descending 6, Sigmoid 21, Rectum 6	II 11, III 6, IV 9	II 11, III 11, IV 11	Niti-S, WallFlex	Dennis
Jun Kawachi [41]	Japan	CS	SEMS/DT	19	12	69.4 ± 12.3	74.1 ± 10.5	8/11	5/7	Splenic flexure 3, Descending 5, Sigmoid 11	Descending 1, Sigmoid 11	II 8, III 7, IV 4	II 7, III 2, IV 3	Niti-S	Create Medic
Chang [42]	China	RCT	SEMS/DT	31	32	65.7 ± 6.7	66.5 ± 6.4	18/13	20/12	Colon 20, Rectum 11	Colon 22, Rectum 11	II 7, III 15, IV 9	II 8, III 17, IV 8		
Zhang [43]	China	CS	SEMS/DT	29	30	64.0 ± 16.3	66.9 ± 11.6	19/10	20/10	Splenic flexure 3, Descending 8, Sigmoid 11, Rectum 7	Splenic flexure 1, Descending 6, Sigmoid 17, Rectum 6	II 0, III 24, IV 5	II 0, III 16, IV 14	Niti-S	Create Medic
Ryuichiro Sato [44]	Japan	CS	SEMS/DT	53	23	70.8 ± 1.7	76.0 ± 2.4	28/25	12/11	Ascending 4, Transverse 11, Descending 11, Sigmoid 20, Rectum 7	Transverse 3, Descending 4, Sigmoid 11, Rectum 5	II 24, III 29	II 10, III 13	Niti-S	Dennis
Yoshiyuki Suzuki [45]	Japan	CS	SEMS/DT	19	21	67.62 ± 6.12	69.25 ± 4.0	8/11	11/10	Ascending 11, Transverse 8	Caecum 4, Ascending 6, Transverse 11	II 9, III 5, IV 5	II 8, III 12, IV 1		
Yin [46]	China	RCT	SEMS/DT	35	35	56.14 ± 16.12	57.12 ± 15.02	20/15	21/14	Descending 9, Sigmoid 21, Other 5	Descending 10, Sigmoid 21, Other 4				
Yue An [21]	China	CS	SEMS/DT	139	67	67.9 ± 12.5	65.9 ± 11.0	85/54	43/24	Descending 39, Sigmoid 61, Junction of Sigmoid and Rectum 16, Rectum 23	Descending 13, Sigmoid 9, Junction of Sigmoid and Rectum 31, Rectum 14	II 27, III 84, IV 28	II 12, III 35, IV 20	WallFlex	Create Medic
Xu [47]	China	CS	SEMS/DT	27	32	65.2 ± 7.3	63.7 ± 12.5	17/10	19/13	Splenic flexure 2, Descending 5, Sigmoid 20	Splenic flexure 3, Descending 5, Sigmoid 24	II 5, III 16, IV 6	II 9, III 18, IV 5	Niti-S	Create Medic
Hiroyuki Inoue [48]	Japan	CS	SEMS	23	25			12/11	8/17	Right 11, Left 20, Rectum 17				WallFlex, Hanaro, Naturfit, Niti-S	Dennis
Akihiro Kondo [49]	Japan	CS	SEMS/DT	65	133	73 ± 3	72 ± 3.2	46/19	82/51	Left 58, Rectum 7	Left 99, Rectum 34				
Kentaro Sato [50]	Japan	CS	SEMS/DT	60	18	69.5 ± 9.0	66.0 ± 9.25	31/29	8/10	Left 38, Rectum 22	Left 10, Rectum 8			WallFlex, Niti-S, Naturfit	Dennis
Zhang [51]	China	CS	SEMS/DT	32	30	63.2 ± 14.2	64.8 ± 10.4	20/12	18/12	Descending 11, Sigmoid 15, Rectum 6	Descending 5, Sigmoid 16, Rectum 9	B 16, C 16	B 16, C 14	Niti-S	Dennis
Okuda [52]	Japan	CS	SEMS/DT	65	115	71 ± 3.0	72 ± 2.2	28/37	49/66	Transverse 14, Descending 14, Sigmoid 37	Transverse 16, Descending 27, Sigmoid 72	II 39, III 26	II 66, III 49	WallFlex, Niti-S	Dennis, Create Medic

*SEMS* self-expanding metal stent, *DT* decompression tube, *RCT* randomized controlled trial, *CS* cohort studies

### Quality assessment results

**Fig. 2:**
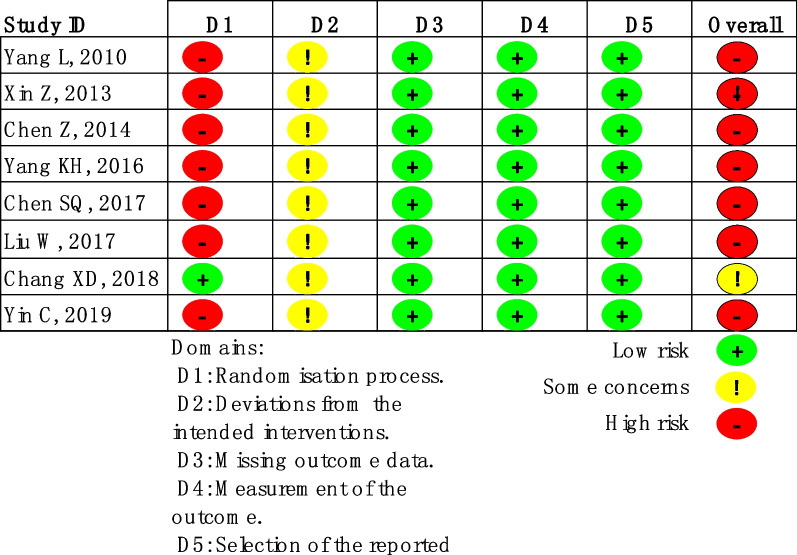
The risk of bias graph for randomized controlled trials based on ROB-2

**Fig. 3:**
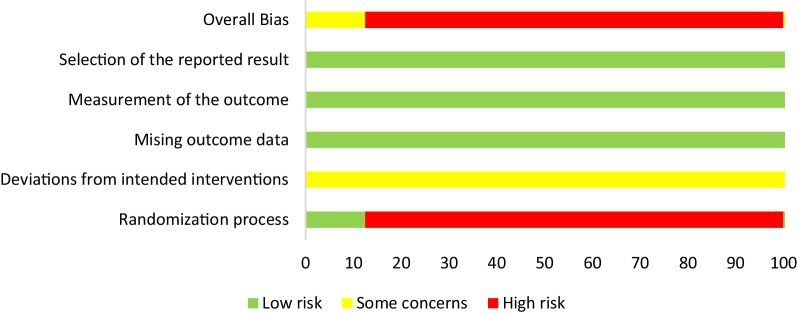
The risk of bias summary for randomized controlled trials based on ROB-2

Within the eight RCTs analyzed, seven were defined at high bias risk, utilizing the Cochrane RoB-2 tool, for inadequate random sequence generation detail (Figs. 2, 3). Due to the study's unique nature, allocation concealment feasibility was limited. All literature sources presented complete, non-selective outcome data. Regarding the eighteen cohort studies, four articles failed to define participant inclusion and exclusion criteria, thus posing significant bias risk based on ROBINS-I assessment (Additional file 3: Fig. S1; Additional file 4: Fig. S2).

### Operation-related outcomes

**Fig. 4:**
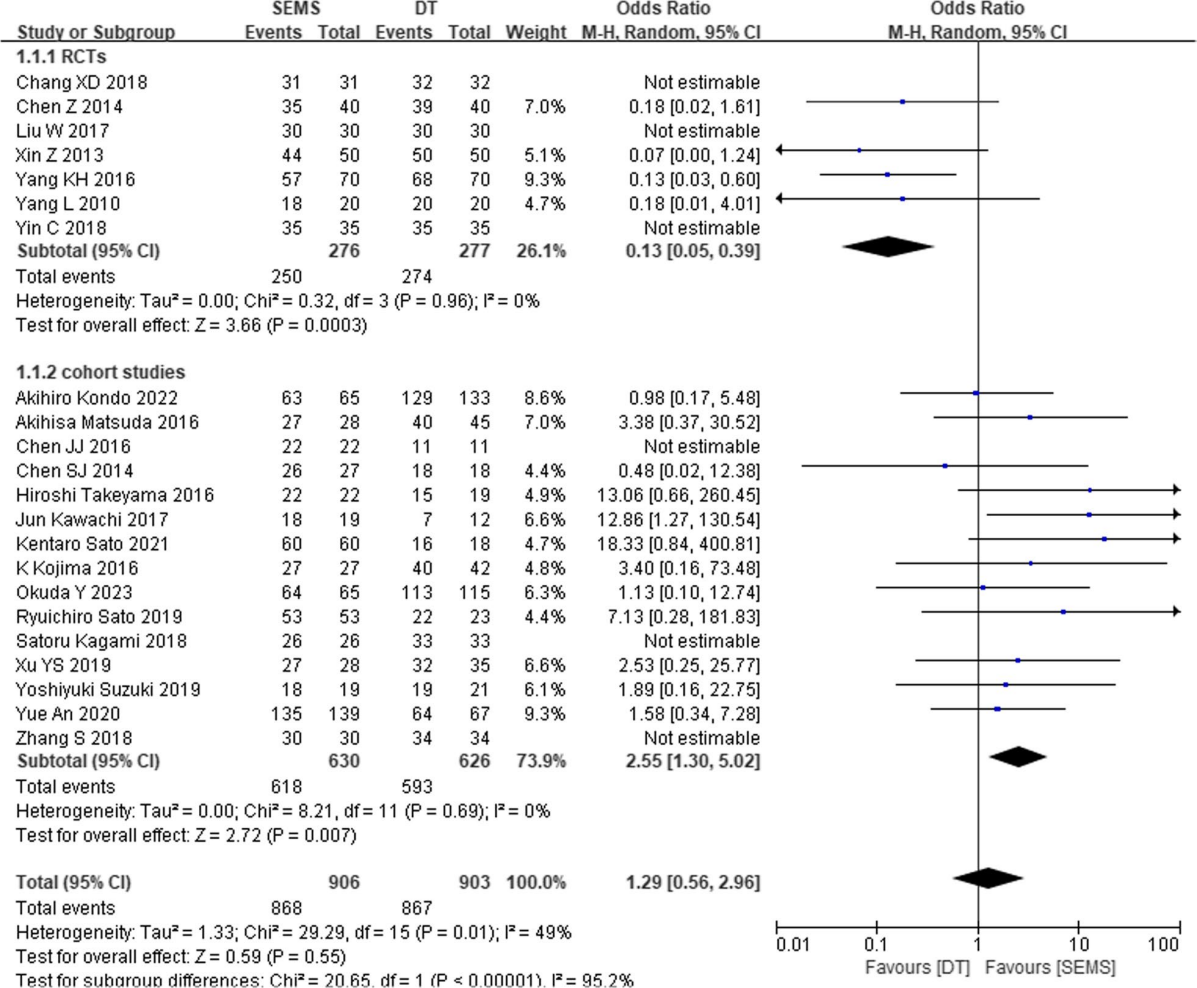
Forest plot of meta-analysis results regarding technical success in SEMS and DT groups. SEMS, self-expanding metal stent; DT, decompression tube; CI, confidence interval; M-H, Mantel–Haenszel; df, degree of freedom

Seven RCTs and fifteen cohort studies reported on SEMS and DT procedural technical success, encompassing 906 SEMS and 903 DT participants (Fig. 4). A random-effects model was applied to pool data. Results indicated no significant difference between the two groups (OR = 1.29, 95% CI 0.56, 2.96, *P* = 0.55). Subgroup analyses were conducted, stratified by study type. DT exhibited superior technical success over SEMS in the RCT subgroup (OR = 0.13, 95% CI 0.05, 0.39, *P* = 0.0003) and SEMS demonstrated superior than DT in the cohort study subgroup (OR = 2.25, 95% CI 1.30, 5.02, *P* = 0.007).

**Fig. 5:**
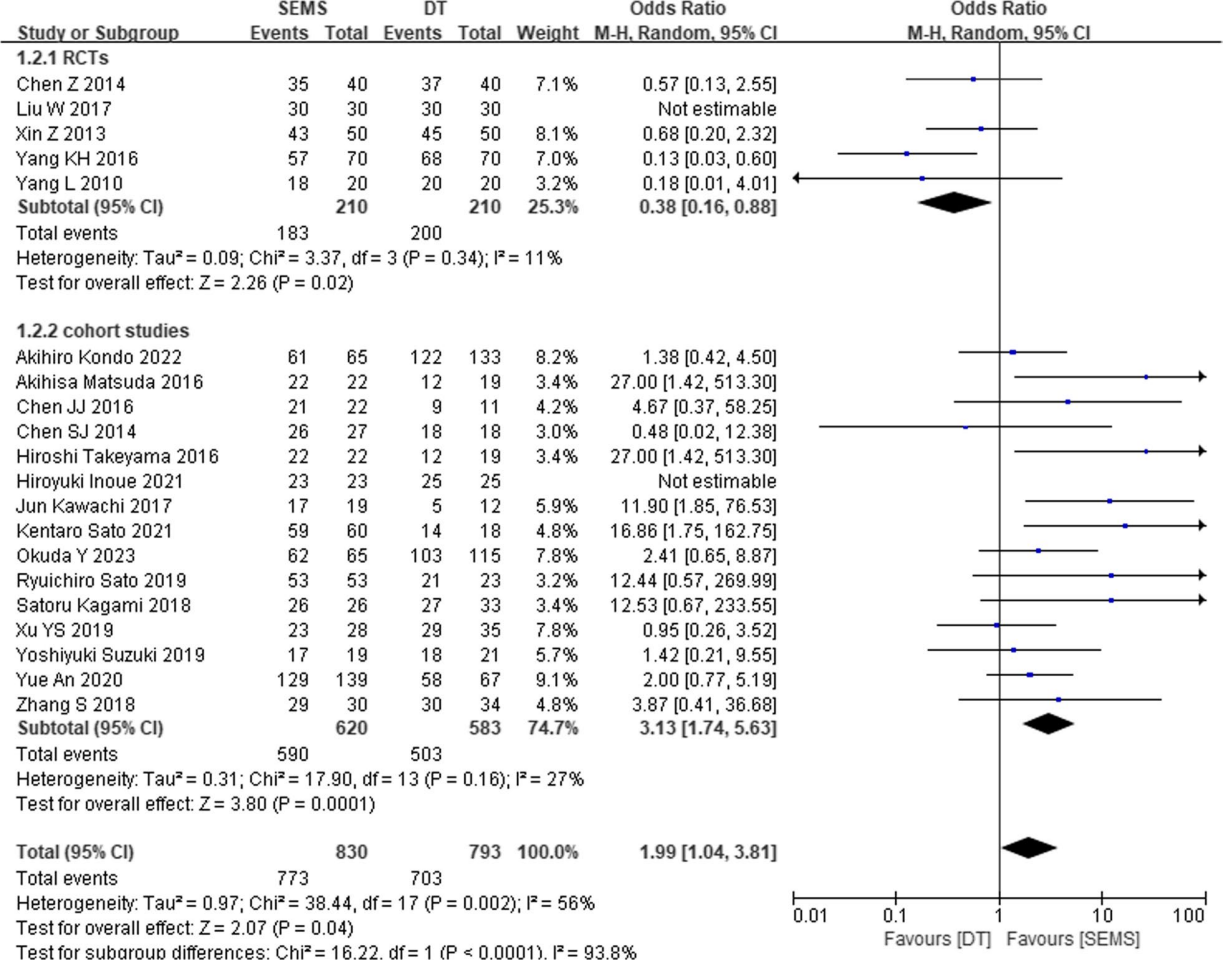
Forest plot of meta-analysis results regarding clinical success in SEMS and DT groups. SEMS, self-expanding metal stent; DT, decompression tube; CI, confidence interval; M-H, Mantel–Haenszel; df, degree of freedom

Five RCTs and fifteen cohort studies reported clinical success rates for SEMS and DT, with 830 SEMS and 703 DT participants (Fig. 5). The results revealed a significantly higher rate of clinical success in the SEMS group than the DT group (OR = 1.99, 95% CI 1.04, 3.81, *P* = 0.04). Stratifying by study type, RCT subgroup revealed higher clinical success rates for DT than SEMS (OR = 0.38, 95% CI 0.16, 0.88, *P* = 0.02). Conversely, in the cohort study subgroup, SEMS was superior over DT (OR = 1.99, 95% CI 1.04, 3.81, *P* = 0.04).

Three cohort studies reported on operation-related abdominal pain post SEMS and DT placement, the SEMS group encompassed 102 participants, while the DT group included 52 (Additional file 5: Fig. S3). Results signified significantly reduced abdominal pain incidence in the SEMS group compared to the DT group (OR = 0.16, 95% CI 0.05, 0.50, *P* = 0.002).

### Surgery-related outcomes

**Fig. 6:**
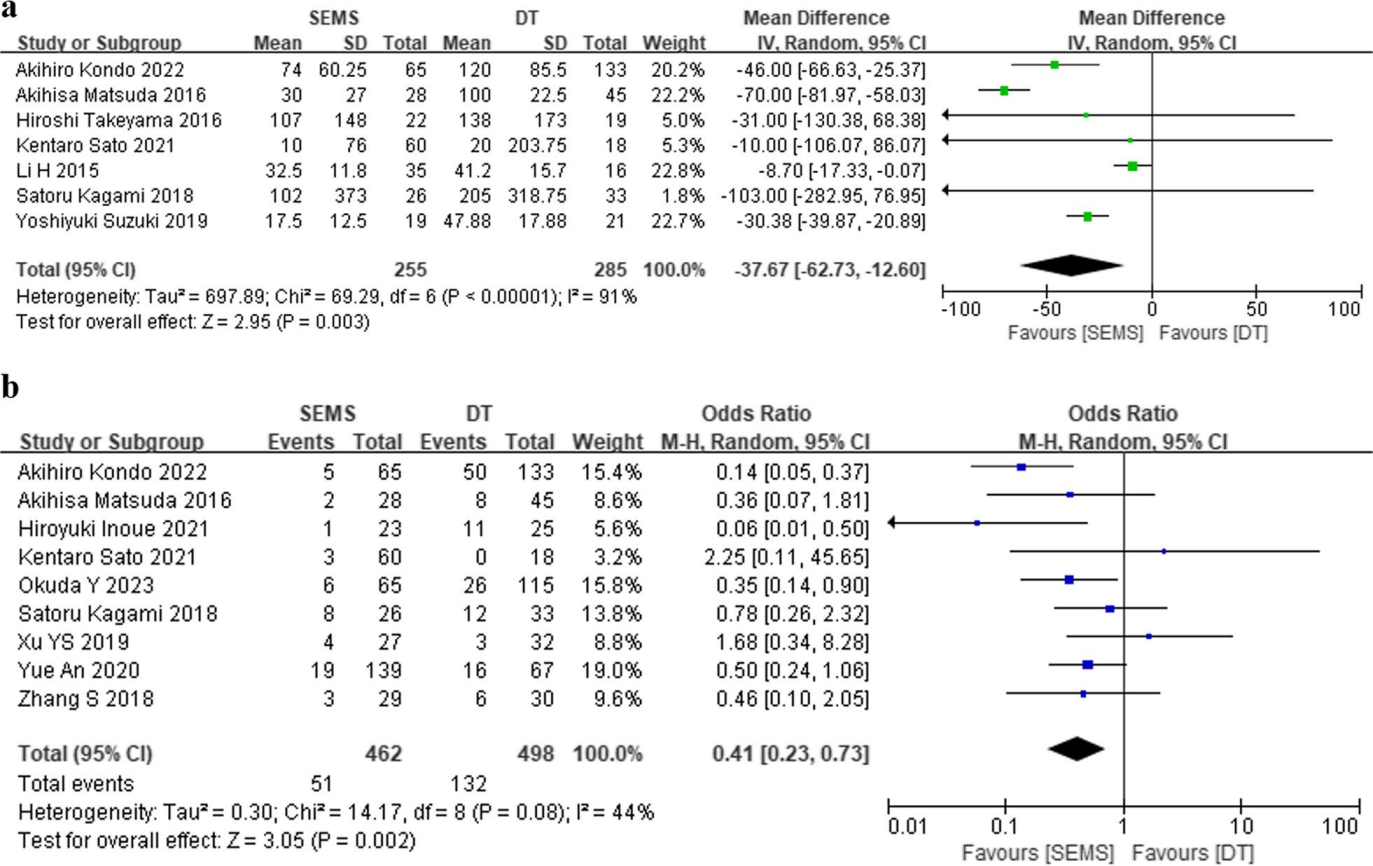
Forest plot of meta-analysis results regarding intraoperative bleeding (**a**) and stoma creation (**b**) in SEMS and DT groups. SEMS, self-expanding metal stent; DT, decompression tube; CI, confidence interval; M-H, Mantel–Haenszel; df, degree of freedom

Among seven cohort studies comparing intraoperative bleeding, SEMS comprised 255 participants and DT comprised 285 (Fig. 6a). Outcomes revealed significantly less intraoperative bleeding in the SEMS group (MD = − 37.67, 95% CI − 62.73, − 12.60, *P* = 0.003). For post-surgery stoma creation, nine cohort studies included 462 SEMS participants and 498 DT participants (Fig. 6b). Outcomes indicated a lower stoma creation rate in the SEMS group compared to the DT group (OR = 0.41, 95% CI 0.23, 0.73, *P* = 0.002).

**Fig. 7:**
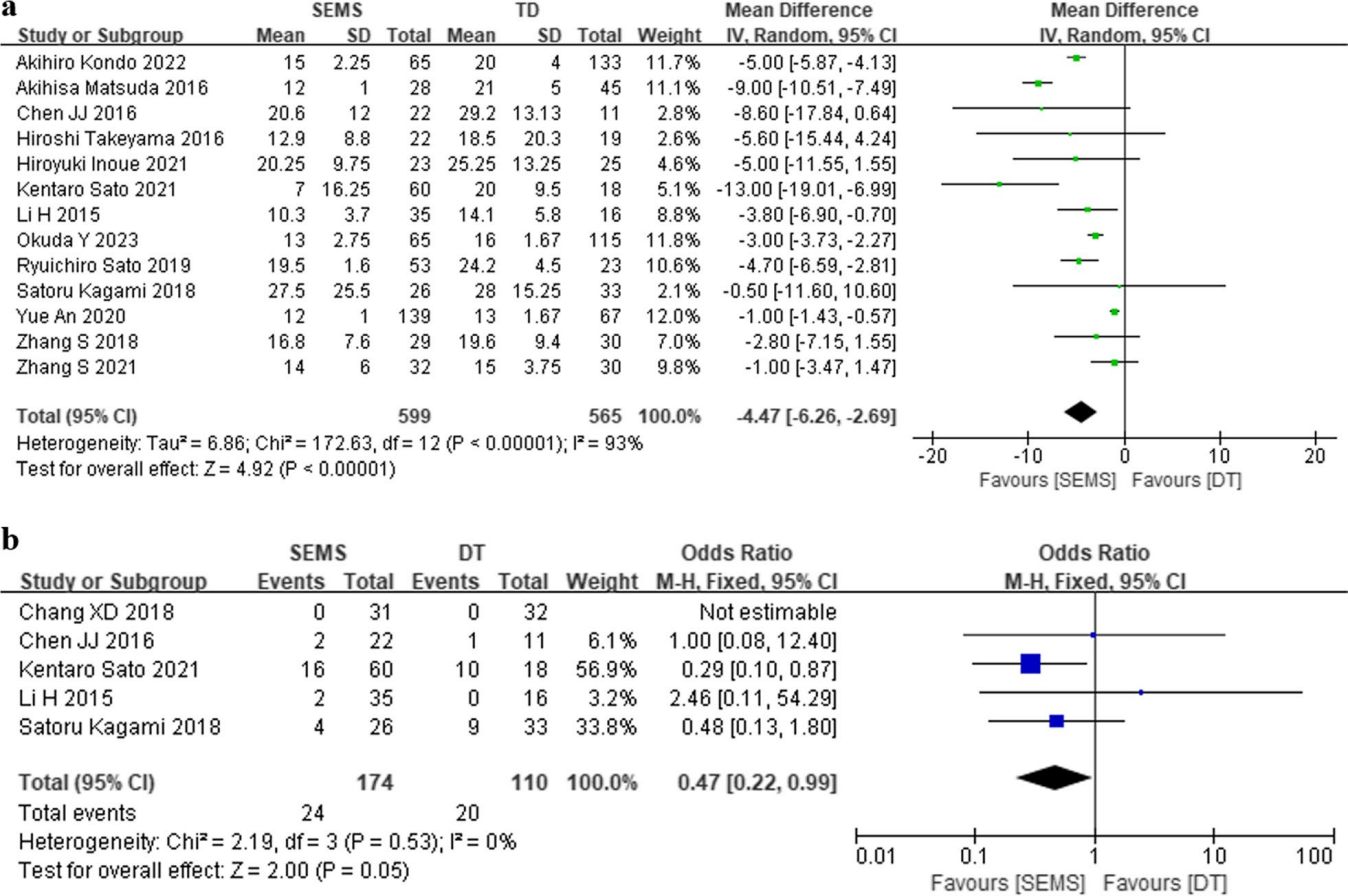
Forest plot of meta-analysis results regarding postoperative hospital stay (**a**) and tumor recurrence (**b**) in SEMS and DT groups. SEMS, self-expanding metal stent; DT, decompression tube; CI, confidence interval; M-H, Mantel–Haenszel; df, degree of freedom

Thirteen cohort studies were included to compared SEMS and DT groups postoperative hospital stay (Fig. 7a). The SEMS group included 599 participants, while the DT group 565. Outcomes indicated a shorter postoperative length of hospital stays in the DT group compared to the SEMS group (MD = − 4.47, 95% CI − 6.26, − 2.69, *P* < 0.00001).

### Long-term outcomes

Tumor recurrence was reported in one RCT and four cohort studies, including 174 SEMS participants and 110 DT participants (Fig. 7b). The result indicated reduced tumor recurrence rates in the SEMS group compared to the DT group (OR = 0.47, 95% CI 0.22, 0.99, *P* = 0.05).

However, no statistically significant differences were observed between the utilization of SEMS and DT in the context of colorectal cancer obstruction, with respect to operation-related perforation (OR = 0.56, 95% CI 0.29, 1.05, *P* = 0.07), device migration (OR = 0.56, 95% CI 0.23, 1.37, *P* = 0.20), postoperative anastomotic leakage (OR = 1.11, 95% CI 0.61, 2.00, *P* = 0.74), postoperative infection (OR = 0.77, 95% CI 0.42, 1.41, *P* = 0.39), postoperative 30-day mortality (OR = 0.62, 95% CI 0.20, 1.91, *P* = 0.40), overall survival rate (OR = 0.91, 95% CI 0.40, 2.04, *P* = 0.81), recurrence-free rates (OR = 1.32, 95% CI 0.81, 2.17, *P* = 0.27), and tumor metastasis (OR = 0.46, 95% CI 0.20, 1.08, *P* = 0.07). Further details are available in the Additional file 6.

### Sensitivity analysis

A sensitivity analysis evaluated the robustness of SEMS and DT meta-analysis results concerning intraoperative bleeding (I2 = 91%) and postoperative hospital stay duration (I2 = 93%), with high study heterogeneity. A literature exclusion approach was employed. Sequentially excluding individual studies resulted in unchanged outcomes, validating meta-analysis reliability.

### GRADE evidence

Outcome indicators were graded individually for RCTs and cohort studies, aligned with GRADE evidence levels (Table 2). Due to blinding challenges, outcomes require cautious interpretation. Among RCT-derived indicators, technical success and clinical success evidence levels were moderate, while operation-related perforation, postoperative anastomotic leakage, and infection evidence levels were low. Cohort study-derived indicators showcased low tumor metastasis evidence levels, and very low evidence levels for technical success, clinical success, operation-related perforation, operation-related abdominal pain, device migration, intraoperative bleeding, postoperative stoma creation, postoperative hospital stays, postoperative anastomotic leakage, postoperative infection, postoperative 30-day mortality, overall survival, recurrence-free rate, and tumor recurrence.

**Table 2:** Certainty assessment of evidence

Quality assessment							No of patients		Effect		Certainty	Importance
No of studies	Design	Risk of bias	Inconsistency	Indirectness	Imprecision	Other considerations	SEMS	DT	Relative (95% CI)	Absolute		
*Technical success*												
7	Randomised trials	Serious^1^	No serious inconsistency	No serious indirectness	No serious imprecision	None	250/276 (90.6%)	274/277 (98.9%)	OR 0.13 (0.05 to 0.39)	67 fewer per 1000 (from 16 to 169 fewer)	MODERATE	CRITICAL
*Technical success*												
15	Observational studies	Serious^1^	No serious inconsistency	No serious indirectness	No serious imprecision	None	618/630 (98.1%)	526/593 (88.7%)	OR 2.55 (1.30 to 5.02)	65 more per 1000 (from 24 to 88 more)	VERY LOW	CRITICAL
*Clinical success*												
5	Randomised trials	Serious^1^	No serious inconsistency	No serious indirectness	No serious imprecision	None	183/210 (87.1%)	200/210 (95.2%)	OR 0.38 (0.16 to 0.88)	69 fewer per 1000 (from 6 to 190 fewer)	MODERATE	CRITICAL
*Clinical success*												
15	Observational studies	Serious^1^	No serious inconsistency	No serious indirectness	No serious imprecision	None	590/620 (95.2%)	503/583 (86.3%)	OR 3.13 (1.74 to 5.63)	89 more per 1000 (from 53 to 110 more)	VERY LOW	CRITICAL
Operation-related perforation												
2	Randomised trials	Serious^1^	No serious inconsistency	No serious indirectness	Serious^3^	None	1/55 (1.8%)	0/55 (0%)	OR 3.15 (0.12 to 82.16)	-	LOW	IMPORTANT
*Operation-related perforation*												
12	Observational studies	Serious^1^	No serious inconsistency	No serious indirectness	No serious imprecision	None	11/585 (1.9%)	28/548 (5.1%)	OR 0.51 (0.26 to 0.99)	24 fewer per 1000 (from 0 to 37 fewer)	VERY LOW	IMPORTANT
*Operation-related abdominal pain*												
3	Observational studies	Serious^1^	No serious inconsistency	No serious indirectness	No serious imprecision	None	8/102 (7.8%)	16/52 (30.8%)	OR 0.16 (0.05 to 0.5)	241 fewer per 1000 (from 126 to 286 fewer)	VERY LOW	IMPORTANT
*Device migration*												
8	Observational studies	Serious^1^	No serious inconsistency	No serious indirectness	Serious^3^	None	6/363 (1.7%)	12/375 (3.2%)	OR 0.56 (0.23 to 1.37)	14 fewer per 1000 (from 24 fewer to 11 more)	VERY LOW	IMPORTANT
*Intraoperative bleeding*												
7	Observational studies	Serious^1^	Serious^2^	No serious indirectness	Serious^3^	None	255	285	-	MD 37.67 lower (62.73 to 12.6 lower)	VERY LOW	IMPORTANT
*Postoperative stoma creation*												
9	Observational studies	Serious^1^	Serious^2^	No serious indirectness	No serious imprecision	None	51/462 (11%)	132/498 (26.5%)	OR 0.41 (0.23 to 0.73)	136 fewer per 1000 (from 57 to 188 fewer)	VERY LOW	IMPORTANT
*Postoperative hospital stays*												
13	Observational studies	Serious^1^	Serious^2^	No serious indirectness	No serious imprecision	None	599	565	-	MD 4.47 lower (6.26 to 2.69 lower)	VERY LOW	IMPORTANT
*Postoperative anastomotic leakage*												
5	Randomised trials	Serious^1^	No serious inconsistency	No serious indirectness	Serious^3^	None	3/171 (1.8%)	2/172 (1.2%)	OR 1.61 (0.25 to 10.34)	7 more per 1000 (from 9 fewer to 97 more)	LOW	IMPORTANT
*Postoperative anastomotic leakage*												
13	Observational studies	Serious^1^	No serious inconsistency	No serious indirectness	Serious^3^	None	19/585 (3.2%)	20/534 (3.7%)	OR 1.06 (0.57 to 1.98)	2 more per 1000 (from 16 fewer to 34 more)	VERY LOW	IMPORTANT
*Postoperative infection*												
7	Randomised trials	Serious^1^	No serious inconsistency	No serious indirectness	Serious^3^	None	10/291 (3.4%)	9/292 (3.1%)	OR 1.15 (0.44 to 2.99)	4 more per 1000 (from 17 fewer to 56 more)	LOW	IMPORTANT
*Postoperative infection*												
8	Observational studies	No serious risk of bias	No serious inconsistency	No serious indirectness	Serious^3^	None	11/246 (4.5%)	15/253 (5.9%)	OR 0.58 (0.26 to 1.29)	24 fewer per 1000 (from 43 fewer to 16 more)	VERY LOW	IMPORTANT
*Postoperative 30d mortality*												
7	Observational studies	Serious^1^	No serious inconsistency	No serious indirectness	Serious^3^	None	4/358 (1.1%)	7/410 (1.7%)	OR 0.62 (0.20 to 1.91)	6 fewer per 1000 (from 14 fewer to 15 more)	VERY LOW	CRITICAL
*Overall survival*												
6	Observational studies	No serious risk of bias	No serious inconsistency	No serious indirectness	Serious^3^	None	151/208 (72.6%)	178/246 (72.4%)	OR 0.91 (0.40 to 2.04)	19 fewer per 1000 (from 212 fewer to 119 more)	VERY LOW	IMPORTANT
*Recurrence free*												
7	Observational studies	Serious^1^	No serious inconsistency	No serious indirectness	No serious imprecision	None	205/311 (65.9%)	226/368 (61.4%)	RR 1.32 (0.81 to 2.17)	197 more per 1000 (from 117 fewer to 719 more)	VERY LOW	IMPORTANT
*Tumor recurrence*												
4	Observational studies	Serious^1^	No serious inconsistency	No serious indirectness	No serious imprecision	None	24/143 (16.8%)	20/78 (25.6%)	RR 0.47 (0.22 to 0.99)	136 fewer per 1000 (from 3 to 200 fewer)	VERY LOW	IMPORTANT
*Tumor metastasis*												
3	Observational studies	No serious risk of bias	No serious inconsistency	No serious indirectness	No serious imprecision	None	19/117 (16.2%)	12/45 (26.7%)	RR 0.50 (0.16 to 1.62)	133 fewer per 1000 (from 224 fewer to 165 more)	LOW	IMPORTANT

*CI* Confidence interval, *MD* mean difference, *RR* risk ratio

## Discussion

This systematic review and meta-analysis enrolled twenty-six studies involving 2,061 participants with colorectal cancer obstruction. The results of the analysis indicated that SEMS had advantages over DT in several aspects of managing colorectal cancer obstruction, such as clinical success, operation-related abdominal pain, intraoperative bleeding, stoma creation, length of postoperative hospital stay, and long-term tumor recurrence rate. However, the two methods were not significantly different in terms of technical success, operation-related perforation, device migration, postoperative anastomotic leakage, postoperative infection rate, 30-day mortality rate, survival rate, recurrence-free rate, and tumor metastasis.

SEMS are delivered via a stent placement system to the lesion to dilate the intestine and relieve the obstruction. The internal diameter of a dilated SEMS is in the range of 18–25 mm [53]. DT, with an internal diameter of about 7 mm, is fixed by an inflated balloon catheter before the obstructive lesion [54]. The application of SEMS or DT as a BTS effectively relieves symptoms in patients with colorectal cancer obstruction, avoiding the need for emergency surgery. In this study, SEMS had a higher clinical success rate than that of DT (98.3% vs. 77.8%, *P* = 0.009). Xu et al. [55] reached the same conclusion on the effectiveness of SMES and DT for left-sided colon obstruction. There are fewer clinical studies of SEMS and DT for the treatment of patients with right-sided colon cancer obstruction. In a study by Yoshiyuki Suzuki et al. [45]. the technical and clinical success rates for SEMS for right-sided colon cancer obstruction were 94.7% and 89.5%, respectively, and for DT 90.5% and 85.7%, respectively, which were not significantly difference. Analyzing the subgroups according to the different sites of obstruction, we found that the clinical success rate of SEMS was higher than DT for obstructions in any part of the colon and showed a trend to be higher than DT in the left-side group. Therefore, for left-sided colon cancer obstruction, it is more advantageous to use SEMS as a bridge to surgery.

However, sub-group analyses based on different study designs indicate that the effects are different between pooled RCTs and cohort studies. The underlying cause may potentially be attributed to the temporal orientation of the data derived from the RCTs, before the year 2014. During this period, DT was notably more prevalent for addressing colorectal cancer obstructions. There is a substantial change in 2012, when SEMS was included in the reimbursement list in Japan and the relevant researches surged. At the same time, the evolution in endoscopic technology and stent material bolstered the effectiveness of SEMS in colorectal cancer obstructions. Therefore, we performed further analyses based on the timing of the studies and found that there was no significant difference between the clinical success rates of SEMS and DT before 2014 (OR = 0.75, 95% CI 0.23, 2.42, *P* = 0.63), whereas SEMS was superior than DT after 2014 (OR = 2.97, 95% CI 1.61, 5.50, *P* = 0.0005). Possible reasons for these observations are: (1) the larger internal diameter of the SEMS makes it easier for feces to pass; (2) the smaller diameter of the DT is prone to blockage, which affects obstruction relief; and (3) after placement of the DT, it requires medical professionals for long-term maintenance to flush and drain it, and the risk of artificially caused decompression failure is high.

Operation-related abdominal pain is one of the common complications after endoscopic placement of SEMS and DT. In a study by Chen et al. [36] on intestinal stents and intestinal obstruction tubes for acute left-sided colorectal cancer obstruction, the incidence of abdominal pain in the DT group was as high as 72.7% (8/11) compared with 18.2% (4/22) in the SEMS group. The occurrence of abdominal pain caused by intestinal obstruction tubes might be due to the following: (1) during maintenance, it is necessary to dilute the stool by injecting water and other solvents through the tube into the obstructed intestinal lumen, which briefly causes an increase in pressure in the intestinal lumen; (2) because one end of the tube is fixed to the obstructing lesion and the other end is connected to a suction device, a mechanical force pulls the intestinal wall; and (3) after the successful placement of the intestinal obstruction tube, the tip of the tube protrudes and compresses the intestinal wall, which increases the probability of abdominal pain, and in severe cases, ischemic necrosis of the intestinal wall may occur, causing intestinal perforation. Perforation is the most common and serious complication of endoscopic operations and often requires emergency surgery. In this study, the incidence rates of operation-related perforations were 1.9% and 4.6% for SEMS and DT, respectively (*P* = 0.07). The risk of stent-related perforation is significantly increased in patients receiving adjunctive chemotherapy, particularly anti-angiogenic agents, with those receiving bevacizumab therapy having a higher risk than that of patients not receiving chemotherapy [57].

With the development of laparoscopic technology, laparoscopic surgery has become the preferred method for the treatment of colorectal cancer because of its advantages such as accurate identification of the lesion site, small surgical trauma, and fast postoperative recovery. However, laparoscopic surgery should be avoided for patients with severe intestinal dilation and edema [58, 59]. In a study conducted by Sato et al. [50] on the treatment of obstructive colorectal cancer with SEMS and DT, the rates of laparoscopic surgery were 100% (60/60) and 44.4% (8/18), respectively (*P* < 0.001). Matsuda et al. [33] reported a laparoscopic surgery rate of 96.4% (27/28) in the SEMS group, whereas the DT group had a rate of only 2.2% (1/45) (*P* < 0.001). These results suggest that SEMS is more effective in relieving intestinal obstruction and bowel preparation, improving bowel dilation and edema, and is suitable for laparoscopic surgery, resulting in less intraoperative bleeding, lower incidence of stoma creation, and shorter length of postoperative hospital stay.

The long-term impact of SEMS and DT as a BTS for patients with obstructive colorectal cancer remains unclear. In a retrospective study by Takahashi et al. [60] comparing the differences in tumor biology between SEMS and DT as a BTS for obstructive colorectal cancer, the SEMS group showed significantly higher plasma concentrations of cell-free DNA than did the DT group (992 vs. 308 ng/mL, *P* = 0.005). Similarly, circulating tumor DNA was higher in the SEMS group than in the DT group (83% vs. 22%, *P* = 0.002). However, in a study by Okuda et al. [52], no significant differences in 5-year survival and 5-year disease-free survival in patients with stage II/III non-right colorectal cancer were found between SEMS and DT placement (83.7% vs. 86.4%, *P* = 0.822 and 64.7% vs 66.4%, *P* = 0.854, respectively). In the current study, the long-term outcomes of survival rate, recurrence-free rate, and tumor metastasis were also not significantly different. However, the tumor recurrence rate was lower in the SEMS group than in the DT group. Given the small sample size and retrospective nature of the included studies, further large-scale, multicenter, high-quality RCTs are needed to validate these findings.

The limitations of this study are as follows. (1) We included twenty-six eligible studies, all of which were from Asian countries. This geographical variation may introduce clinical heterogeneity and affect the generalizability of our results. (2) The included studies involved participants with different types of obstructions caused by colorectal cancer, with one study focusing on right-sided obstructions, six studies on obstructions in any part of the colon, and the remaining studies on left-sided obstructions. This variation in patient characteristics may have resulted in baseline differences among the patients. (3) The included studies used different SEEMS/DT models, which may serve as a confounding factor in our study. (4) Owing to significant bias, the certainty level of the evidence is not very high.

## Conclusion

Both SEMS and DT are effective as BTS when treating obstructions due to colorectal cancer. However, the analysis results indicate that SEMS is better than DT at managing colorectal cancer obstruction, such as clinical success, operation-related abdominal pain, intraoperative bleeding, stoma creation, length of postoperative hospital stay, and long-term tumor recurrence. Therefore, as a BTS, SEMS should be the preferred option for patients with colorectal cancer obstruction. Further large-scale international clinical trials are still needed to verify the efficacy of both SEMS and DT for colorectal cancer obstruction in different countries.

### Supplementary Information

**Additional file 1**: **Table S1**. Electronic search strategies.

**Additional file 2**: **Table S2**. Detailed information on included studies.

**Additional file 3**: **Fig S1**. The risk of bias graph for cohort studies based on ROBINS-I.

**Additional file 4**: **Fig S2**. The risk of bias summary for cohort studies based on ROBINS-I.

**Additional file 5**: **Fig S3**. Forest plot of meta-analysis results regarding operation-related abdominal pain in SEMS and DT groups. SEMS, self-expanding metal stent; DT, decompression tube; CI, confidence interval; M-H, Mantel-Haenszel; df, degree of freedom.

**Additional file 6**: Other research findings.

## Data Availability

All data generated or analyzed during this study are included in this published article and its supplementary information files.

## Abbreviations

BTS: Bridge to surgery
CI: Confidence interval
CT: Computed tomography
DT: Decompression tube
ESGE: European Society of Gastrointestinal Endoscopy
GRADE: Graded Recommendations Assessment, Development, and Evaluation
MD: Mean difference
OR: Odds ratio
PRISMA: Preferred Reporting Items for Systematic Reviews and Meta-Analyses
RCT: Randomized controlled trial
RoB 2: Cochrane Risk of Bias tool version 2
ROBINS-I: Risk of Bias in Nonrandomized Studies of Interventions
SEMS: Self-expanding metal stent
