# Supplementary material for: Self-expanding metal stents versus decompression tubes as a bridge to surgery for patients with obstruction caused by colorectal cancer: a systematic review and meta-analysis
Source: World J Emerg Surg. 2023 Sep 27;18:46. doi: 10.1186/s13017-023-00515-6 (PMC10536785; doi:10.1186/s13017-023-00515-6)
Supplement: Supplementary file 1 — Additional file 1: Table S1. Electronic search strategies. [file 13017_2023_515_MOESM1_ESM.docx]

Table S1.Electronic Search Strategies

| Database | Search Strategy | Number |
| --- | --- | --- |
| Pubmed | ( ( ( “self expandable metallic stent”[Title/Abstract] ）OR ( “Self Expandable Metal Stent”[Title/Abstract] ) OR ( “Self Expandable Metal Stents”[Title/Abstract] ) OR ( “Self Expandable Metallic Stents”[Title/Abstract] ) OR ( “self-expanding metallic stent”[Title/Abstract] ) OR ( “self-expanding metallic stents”[Title/Abstract] ) OR ( “self-expanding metal stent”[Title/Abstract] ) OR ( “self-expanding metal stents”[Title/Abstract] ) OR ( “ileus tube”[Title/Abstract] ) OR ( “transanal tube”[Title/Abstract] ) OR ( “rectal tube”[Title/Abstract] ) OR ( “anal tube”[Title/Abstract] ) OR ( “decompression tube”[Title/Abstract] ) OR ( “colorectal tube”[Title/Abstract] ) OR ( “colonal tube”[Title/Abstract] ) ) AND ( ( “Colorectal Neoplasm”[Title/Abstract] ) OR ( “Neoplasm, Colorectal”[Title/Abstract] ) OR ( “Neoplasms, Colorectal”[Title/Abstract] ) OR ( “Colorectal Tumors”[Title/Abstract] ) OR ( “Colorectal Tumor”[Title/Abstract] ) OR ( “Tumor, Colorectal”[Title/Abstract] ) OR ( “Tumors, Colorectal”[Title/Abstract] ) OR ( “Colorectal Cancer”[Title/Abstract] ) OR ( “Cancer, Colorectal”[Title/Abstract] ) OR ( “Cancers, Colorectal”[Title/Abstract] ) OR ( “Colorectal Cancers”[Title/Abstract] ) OR ( “Colorectal Carcinoma”[Title/Abstract] ) OR ( “Carcinoma, Colorectal”[Title/Abstract] ) OR ( “Colorectal Carcinomas”[Title/Abstract] ) ) AND ( ( “Intestinal Obstructions”[Title/Abstract] ) OR ( “Obstruction, Intestinal”[Title/Abstract] ) )AND (2000-2022[pdat] )) | 651 |
| EmBase | #1'colorectal cancer':ab,ti,kw OR 'colorectal carcinoma':ab,kw,ti OR 'colorectal tumor':ab,kw,ti | 167 |
|  | #2'self expandable metallic stent':ab,kw,ti OR 'intestinal decompression tube':kw,ti,ab OR 'rectal tube':ab,kw,ti OR 'transanal tube':ab,kw,ti OR 'ileus tube':ab,kw,ti |  |
|  | #3 #1 AND #2 |  |
|  | #4 #1 AND #2 AND [2000-01-01,2022-10-31]/py |  |
| Web of Science | ( ( ( self expandable metallic stent ）OR ( Self Expandable Metal Stent ) OR ( Self Expandable Metal Stents ) OR ( Self Expandable Metallic Stents ) OR ( self-expanding metallic stent ) OR ( self-expanding metallic stents ) OR ( self-expanding metal stent ) OR ( self-expanding metal stents ) OR ( ileus tube ) OR ( transanal tube ) OR ( rectal tube ) OR ( anal tube ) OR ( decompression tube ) OR ( colorectal tube ) OR ( colonal tube ) ) AND ( ( Colorectal Neoplasm ) OR ( Neoplasm, Colorectal ) OR ( Neoplasms, Colorectal ) OR ( Colorectal Tumors ) OR ( Colorectal Tumor ) OR ( Tumor, Colorectal ) OR ( Tumors, Colorectal ) OR ( Colorectal Cancer ) OR ( Cancer, Colorectal ) OR ( Cancers, Colorectal ) OR ( Colorectal Cancers ) OR ( Colorectal Carcinoma ) OR ( Carcinoma, Colorectal ) OR ( Colorectal Carcinomas ) ) AND ( ( Intestinal Obstructions ) OR ( Obstruction, Intestinal ) ) ) AND (2000-2022 ) | 611 |
| CENTRAL | #1 Self Expandable Metal Stent,#2self expandable metallic stent,#3Self Expandable Metal Stents,#4Self Expandable Metallic Stents,#5self-expanding metallic stent,#6self-expanding metallic stents,#7self-expanding metal stent,#8self-expanding metal stents,#9ileus tube,#10transanal tube,#11rectal tube,#12anal tube,#13decompression tube,#14colorectal tube,#15Colorectal Neoplasm,#16Neoplasms,Colorectal,#17Colorectal Tumors,#18Colorectal Tumor,#19Tumors, Colorectal,#20Colorectal Cancer,#21Colorectal Cancers,#22Colorectal Carcinoma#23Colorectal Carcinomas,#24Intestinal Obstructions,#25Obstruction, Intestinal | 42 |
|  | #26 #1 OR #2 OR #3 OR #4 OR #5 OR #6 OR #7 OR #8 OR #9 OR #10 OR #11 OR #12 OR #13 OR #14 |  |
|  | #27 #15 OR #16 OR #17 OR #18 OR #19 OR #20 OR #21 OR #22 OR #23 |  |
|  | #28 #24 OR #25 |  |
|  | #29 #26 AND #27 AND #28 |  |
| CNKI | （篇关摘: 自膨胀金属支架） OR （ 篇关摘: 肠道金架 ) OR (篇关摘: 结直肠架 ) OR (篇关摘: 肠梗阻导管 ) OR (篇关摘: 肠梗阻减压导 ）AND ((篇关摘:结直肠肿瘤) OR ( 篇关摘:结直肠癌)) AND (肠梗阻) AND 时间：2000-2022) | 150 |
| Wanfang data | (((((摘要=自膨胀金属支架) OR 摘要=肠道金属支架) OR 摘要=结直肠支架) OR 摘要=肠梗阻导管) OR 摘要=肠梗阻减压导管) AND ((摘要=结直肠肿瘤) OR 摘要=结直肠癌) AND （时间：2000-2022) | 493 |
| SinoMed | ("肠梗阻"[摘要:智能]) AND (("结直肠肿瘤"[摘要:智能] OR "结直肠癌"[摘要:智能]) AND (("自膨胀金属支架"[摘要:智能] OR "肠道金属支架"[摘要:智能] OR "结直肠支架"[摘要:智能] OR "肠梗阻导管"[摘要:智能] OR "肠梗阻减压导管"[摘要:智能]) AND 2000-2022[日期])) | 128 |
