# Supplementary material for: Self-expanding metal stents versus decompression tubes as a bridge to surgery for patients with obstruction caused by colorectal cancer: a systematic review and meta-analysis
Source: World J Emerg Surg. 2023 Sep 27;18:46. doi: 10.1186/s13017-023-00515-6 (PMC10536785; doi:10.1186/s13017-023-00515-6)
Supplement: Supplementary file 2 — Additional file 2: Table S2. Detailed information on included studies. [file 13017_2023_515_MOESM2_ESM.docx]

Table S2. Detailed information on included studies

| Study | Sample size（SEMS/DT） | study timing | Technical success | Clinical success | Postoperative perforation | Postoperative abdominal pain | Device migration | Laparoscopic surgery | Intraoperative bleeding | Stoma creation | Postoperative anastomotic leakage | Postoperative infection | Postoperative hospital stay | Postoperative 30d mortality | Overall survival | Recurrence-free | Tumor recurrence | Tumor metastasis |
| --- | --- | --- | --- | --- | --- | --- | --- | --- | --- | --- | --- | --- | --- | --- | --- | --- | --- | --- |
| Yang L, 2010 | 20/20 | 2007-2009 | 18/20 | 18/20 | 1/0 |  |  |  |  |  | 0/0 | 0/0 |  |  |  |  |  |  |
| Xin Z, 2013 | 50/50 | 2005-2011 | 44/50 | 43/45 |  |  |  |  |  |  | 0/0 | 0/0 |  |  |  |  |  |  |
| Chen SJ, 2014 | 27/18 | 2008-2014 | 26/18 | 26/18 | 0/0 | 3/8 |  |  |  |  | 0/0 | 0/0 |  |  |  |  |  |  |
| Chen Z, 2014 | 40/40 | 2011-2014 | 35/39 | 35/39 |  |  |  |  |  |  | 0/0 | 0/0 |  |  |  |  |  |  |
| Li H, 2015 | 35/16 | 2009-2014 |  |  |  |  |  |  | 32.5±11.8ml/41.2±15.7ml |  | 1/2 | 4/3 | 10.3±3.7d/14.1±5.8d |  |  |  | 2/0 | 4/1 |
| Akihisa Matsuda, 2016 | 28/45 | 2005-2014 | 27/40 | 27/38 | 0/4 |  | 1/1 | 27/1 | 30±27ml/100±22.5ml | 2/8 | 2/2 |  | 12±1d/21±5d | 0/2 |  |  |  |  |
| K Kojima, 2016 | 27/42 | 2009-2016 | 27/40 |  | 2/6 |  | 2/2 |  |  |  |  |  |  |  |  |  |  |  |
| Hiroshi Takeyama, 2016 | 22/19 | 2010-2015 | 22/15 | 22/12 |  |  |  | 19/10 | 107 ± 148 ml/138 ± 173ml |  | 0/0 | 2/0 | 12.9 ± 8.8d/18.5 ± 20.3d |  |  |  |  |  |
| Chen JJ, 2016 | 22/11 | 2006-2012 | 22/11 | 21/9 | 0/0 | 4/8 | 1/1 |  |  |  | 0/0 | 2/1 | 20.6±12d/29.2±13.13d | 0/0 | 11/6 |  | 2/1 | 3/2 |
| Yang KH, 2016 | 70/70 | 2011-2014 | 57/68 |  |  |  |  |  |  |  |  | 5/4 |  |  |  |  |  |  |
| Chen SQ, 2017 | 50/50 | 2014-2017 |  |  |  |  |  |  |  |  |  | 0/0 |  |  |  |  |  |  |
| Liu W, 2017 | 30/30 | 2014-2016 | 30/30 | 30/30 |  |  |  |  |  |  | 0/0 | 0/0 |  |  |  |  |  |  |
| Satoru Kagami, 2018 | 26/33 | 2013-2015 | 26/33 | 26/27 | 0/3 |  | 0/2 | 20/0 | 102±373ml/205±318.75ml | 8/12 |  |  | 27.5±25.5d/28±15.25d |  | 19/22 | 19/17 | 4/9 |  |
| Jun Kawachi, 2017 | 19/12 | 2006-2016 | 18/7 | 17/5 |  |  |  |  |  |  | 2/0 | 0/1 |  | 2/1 |  |  |  |  |
| Chang XD, 2018 | 31/32 | 2013-2017 | 31/32 |  |  |  |  |  |  |  | 3/2 | 5/5 |  |  |  |  | 0/0 |  |
| Zhang S, 2018 | 30/34 | 2013-2017 | 30/34 | 29/30 |  |  | 1/0 | 22/16 |  | 3/6 |  | 1/5 | 16.8±7.6d/19.6±9.4d |  |  |  |  |  |
| Ryuichiro Sato, 2019 | 53/23 | 2009-2018 | 53/22 | 53/21 | 0/1 | 1/0 |  | 11/2 |  |  | 1/0 |  | 19.5 ± 1.6d/24.2 ± 4.5 d | 1/2 | 43/20 | 36/14 |  |  |
| Yoshiyuki Suzuki, 2019 | 19/21 | 2007-2017 | 18/19 | 17/18 | 1/1 |  |  | 19/13 | 17.5±12.5ml/47.88±17.88ml |  |  |  |  | 0/0 | 6/17 | 9/14 |  |  |
| Yin C, 2018 | 35/35 | 2017-2018 | 35/35 |  | 0/0 |  |  | 30/34 |  |  |  |  |  |  |  |  |  |  |
| Yue An, 2020 | 139/67 | 2014-2017 | 135/64 | 129/58 | 1/1 |  | 1/4 |  |  | 19/16 | 0/1 |  | 12.0±1d/13.0±1.67d |  |  |  |  |  |
| Xu YS, 2019 | 27/32 | 2014-2016 | 27/32 | 23/29 | 2/1 |  | 0/0 |  |  | 4/3 | 1/0 | 2/2 |  | 1/0 |  |  |  |  |
| Hiroyuki Inoue, 2021 | 23/25 | 2007-2019 |  | 23/25 |  |  |  | 19/17 |  | 1/11 | 0/1 |  | 20.25±9.75d/25.25±13.25d |  | 20/17 | 14/12 |  |  |
| Akihiro Kondo, 2022 | 65/133 | 2008-2018 | 63/129 | 61/122 | 2/3 |  |  | 38/33 | 74±60.25ml/120±85.5ml | 5/50 | 4/6 |  | 15±2.25d/20±4d | 0/2 | 52/96 | 40/86 |  |  |
| Kentaro Sato, 2021 | 60/18 | 2005-2019 | 60/116 | 59/14 | 0/2 |  |  | 60/10 | 10±76ml/20±203.75ml | 3/0 | 4/0 |  | 7±16.25d/20±9.5d | 0/0 |  | 45/7 | 16/10 | 12/9 |
| Zhang S, 2021 | 32/30 | 2013-2019 |  |  |  |  |  | 18/13 |  |  |  |  | 14±6d /15±3.75d |  |  |  |  |  |
| Okuda Y, 2023 | 65/115 | 2010-2019 | 64/113 | 62/103 | 3/8 |  | 0/2 | 42/50 |  | 6/26 | 4/8 | 0/3 | 13±2.75d/16±1.67d | 0/0 |  | 42/76 |  |  |
