# Supplementary figures and images for: Self-expanding metal stents versus decompression tubes as a bridge to surgery for patients with obstruction caused by colorectal cancer: a systematic review and meta-analysis
Source: World J Emerg Surg. 2023 Sep 27;18:46. doi: 10.1186/s13017-023-00515-6 (PMC10536785; doi:10.1186/s13017-023-00515-6)

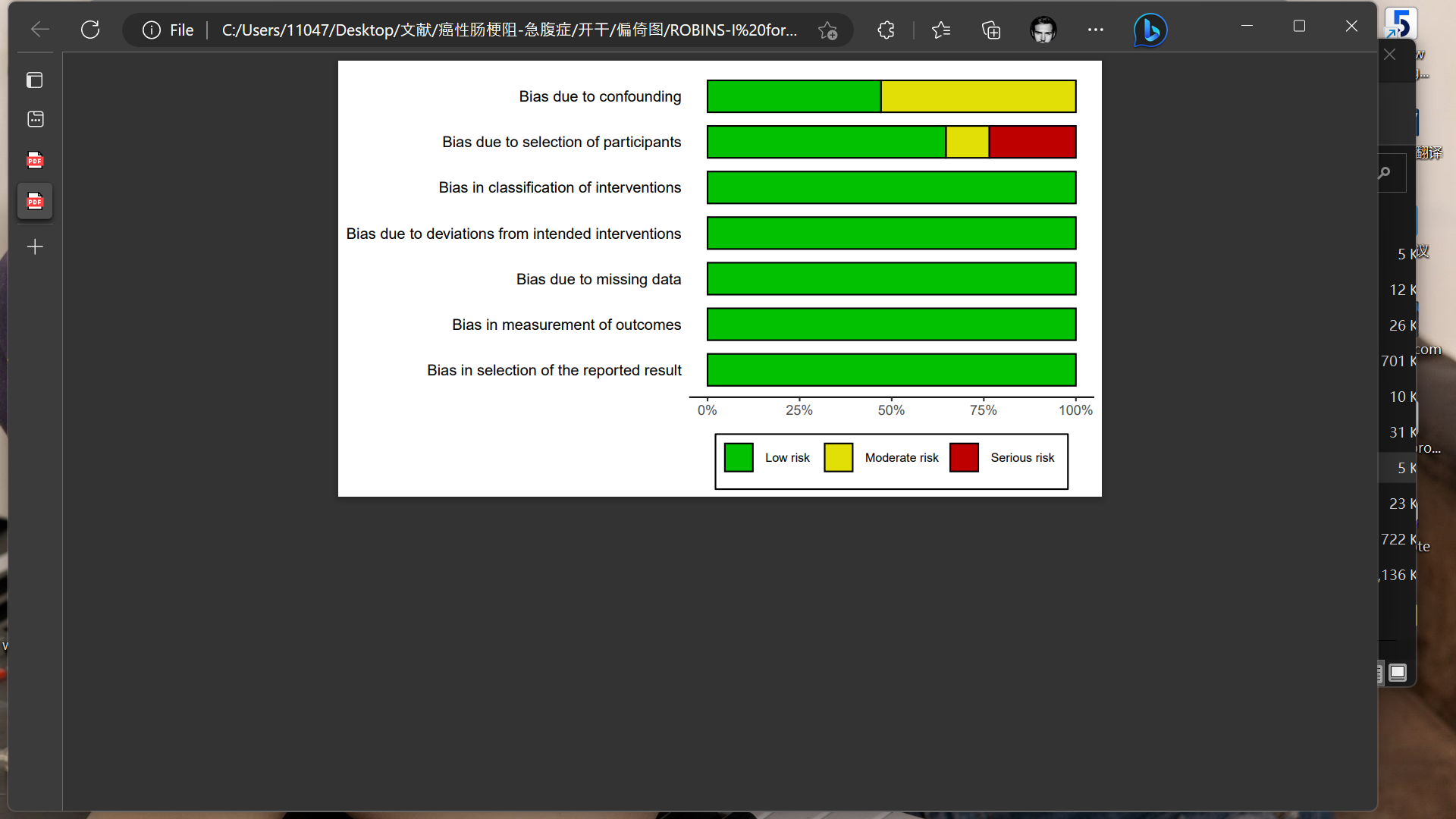

Supplement: Supplementary file 4 — Additional file 4: Fig S2. The risk of bias summary for cohort studies based on ROBINS-I. [file 13017_2023_515_MOESM4_ESM.docx]

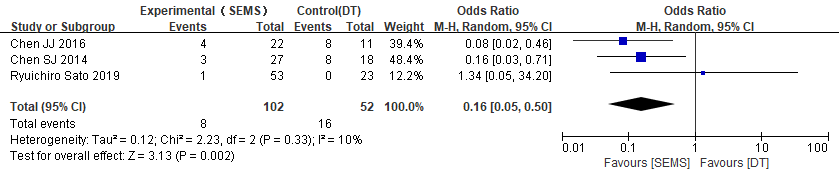

Supplement: Supplementary file 5 — Additional file 5: Fig S3. Forest plot of meta-analysis results regarding operation-related abdominal pain in SEMS and DT groups. SEMS, self-expanding metal stent; DT, decompression tube; CI, confidence interval; M-H, Mantel-Haenszel; df, degree of freedom. [file 13017_2023_515_MOESM5_ESM.docx]
