# Supplementary material for: Self-expanding metal stents versus decompression tubes as a bridge to surgery for patients with obstruction caused by colorectal cancer: a systematic review and meta-analysis
Source: World J Emerg Surg. 2023 Sep 27;18:46. doi: 10.1186/s13017-023-00515-6 (PMC10536785; doi:10.1186/s13017-023-00515-6)
Supplement: Supplementary file 6 — Additional file 6: Other research findings. [file 13017_2023_515_MOESM6_ESM.docx]

Self-expanding metal stents versus decompression tubes as a bridge to surgery for patients with obstruction caused by colorectal cancer: a systematic review and meta-analysis

Wei Ma^1^, Jian-Cheng Zhang^2,3,4^, Kun Luo^1^, Lu Wang^2,3,4^, Chi Zhang^2,3,4^, Bin Cai^2,3,4^ and Hua Jiang^2,3,4^

# Results

**Operation-related outcomes**

A total of [twenty-two](javascript:;) studies, including seven RCTs and [fifteen](javascript:;) cohort studies, reported the technical success of SEMS and DT procedures, involving 906 participants in the SEMS group and 903 participants in the DT group (Fig. 1). There was low heterogeneity among the studies (P=0.01, I^2^=49%), suggesting the adoption of a random-effects model. The results indicated no significant difference between the two groups (OR=1.29, 95% CI: 0.56, 2.96, P=0.55). Among the [twenty-two](javascript:;) included studies, [seventeen](javascript:;) focused on left-sided colon obstructions, one on right-sided colon obstructions, and four on obstructions in any part of the colon. Subgroup analyses revealed no significant differences in the technical success rates between SEMS and DT in the left-sided colon (OR=0.99, 95% CI: 0.36, 2.73, P=0.98), right-sided colon (OR=1.89, 95% CI: 0.16, 22.75, P=0.61), and entire colon (OR=4.03, 95% CI: 0.89, 19.29, P=0.08).

A total of [twenty](javascript:;) studies, including five RCTs and [fifteen](javascript:;) cohort studies, reported the clinical success rates of SEMS and DT, involving 830 participants in the SEMS group and 703 participants in the DT group (Fig. 2). The results revealed a significantly higher rate of clinical success in the SEMS group than in the DT group (OR=1.99, 95% CI: 1.04, 3.81, P=0.04). Among the [twenty](javascript:;) included studies, [seventeen](javascript:;) focused on left-sided colon obstructions, one on right-sided colon obstructions, and two on obstructions in any colon part. Subgroup analyses revealed that for obstruction in any part of the colon, SEMS had a higher clinical success rate than DT (OR=18.64, 95% CI: 2.22, 156.51, P=0.007), whereas no significant differences in clinical success rates were found for left-sided colon obstructions (OR=1.71, 95% CI: 0.86, 3.42, P=0.13) and right-sided colon obstructions (OR=1.42, 95% CI: 0.21, 9.55, P=0.72). Using 2014 as the time point grouping for the study, it was found that there was no significant difference between the clinical success rates of SEMS and DT before 2014 (OR=0.75, 95% CI: 0.23, 2.42, P=0.63), and after 2014, SEMS had better clinical success rates than DT (OR=2.97, 95% CI: 1.61, 5.50, P=0.0005) (Fig.3).

A total of [fourteen](javascript:;) studies, comprising two RCTs and [twelve](javascript:;) cohort studies, reported the incidence of operation-related perforation following SEMS and DT placement, involving 640 participants in the SEMS group and 603 participants in the DT group (Fig. 4). The results indicated no significant difference in the incidence of perforation between the two groups (OR=0.56, 95% CI: 0.29, 1.05, P=0.07). Subgroup analyses by study type showed no difference in operation-related perforation between SEMS and DT in the RCT group (OR=3.15, 95% CI: 0.12, 82.16, P=0.49), whereas operation-related perforation was better in SEMS than in DT in the cohort study group (OR=0.51, 95% CI: 0.26, 0.99, P=0.05).

A total of eight cohort studies were identified reporting the incidence of device migration following SEMS and DT placement, with 363 participants in the SEMS group and 375 participants in the DT group (Fig. 5). The results indicated no significant difference between the two groups (OR=0.56, 95% CI: 0.23, 1.37, P=0.20).

**Surgery-related outcomes**

We identified [eighteen](javascript:;) articles that assessed the occurrence of postoperative anastomotic leakage between the SEMS and DT groups following surgery, comprising five RCTs and [thirteen](javascript:;) cohort studies (Fig. 6). The SEMS group consisted of 756 participants, whereas the DT group comprised 706 participants. The results indicated no significant difference in the occurrence of anastomotic leakage between the two groups (OR=1.11, 95% CI: 0.61, 2.00, P=0.74). Subgroup analysis by study type showed no difference in postoperative anastomotic leakage between SEMS and DT in the RCTs (OR=1.61, 95% CI: 0.25, 10.34, P=0.62) and cohort study groups (OR=1.06, 95% CI: 0.57, 1.98, P=0.86).

The incidence of postoperative infections in the SEMS and DT groups was reported in [fourteen](javascript:;) studies, including seven RCTs and eight cohort studies, with a total of 537 participants in the SEMS group and 545 participants in the DT group (Fig. 7). The results revealed no significant difference in the incidence of postoperative infection between the two groups (OR=0.77, 95% CI: 0.42, 1.41, P=0.39). Subgroup analysis by study type showed no difference in postoperative infection between SEMS and DT in the RCT (OR=1.15, 95% CI: 0.44, 2.99, P=0.78) and cohort study groups (OR=0.58, 95% CI: 0.26, 1.29, P=0.18).

The postoperative 30-day mortality rates in the SEMS and DT groups were examined in nine cohort studies with 358 participants in the SEMS group and 410 participants in the DT group (Fig. 8). The results demonstrated no significant difference in the 30-day mortality rate between the two groups (OR=0.62, 95% CI: 0.20, 1.91, P=0.40).

**Long-term outcomes**

We identified six cohort studies that reported on the overall survival rate following surgery in the SEMS and DT groups, with 208 participants in the SEMS group and 246 participants in the DT group (Fig. 9). The test for heterogeneity indicated a significant heterogeneity among the studies (P=0.02, I^2^=62%); thus, a random-effects model was employed. The results showed no significant difference in the overall survival rate at three years or five years between the two groups (OR=0.91, 95% CI: 0.40, 2.04, P=0.81). Among the six included studies, three reported 3-year overall survival rates, and the other three reported 5-year survival rates. Subgroup analyses revealed no significant differences in 3- or 5-year survival rates between SEMS and DT (OR=1.20, 95% CI: 0.67, 2.15, P=0.54 and OR=0.78, 95% CI: 0.12, 5.00, P=0.80, respectively).

In total, seven cohort studies examined the recurrence-free rates in the SEMS and DT groups, with 311 participants in the SEMS group and 368 participants in the DT group (Fig. 10). The results demonstrated no significant difference in recurrence-free rates between the two groups (OR=1.32, 95% CI: 0.81, 2.17, P=0.27). Among the seven included studies, four reported 3-year recurrence-free rates and three reported 5-year recurrence-free rates. Subgroup analyses revealed no significant differences in 3- or 5-year recurrence-free rates (OR=1.26, 95% CI: 0.54, 2.93, P=0.60 and OR=1.37, 95% CI: 0.75, 2.51, P=0.31, respectively).

Three cohort studies investigated the occurrence of tumor metastasis following surgery in the SEMS and DT groups, with 117 participants in the SEMS group and 45 participants in the DT group (Fig. 11). The results showed no significant difference between the two groups (OR=0.46, 95% CI: 0.20, 1.08, P=0.07).

**Publication bias**

Review Manager 5.4 software assessed the publication bias of SEMS and DT in patients with colorectal cancer obstruction. Taking the technical success and clinical success as an example, the funnel plot showed symmetrical data point distribution around the center line, signifying no significant publication bias among selected studies (Fig. 12).


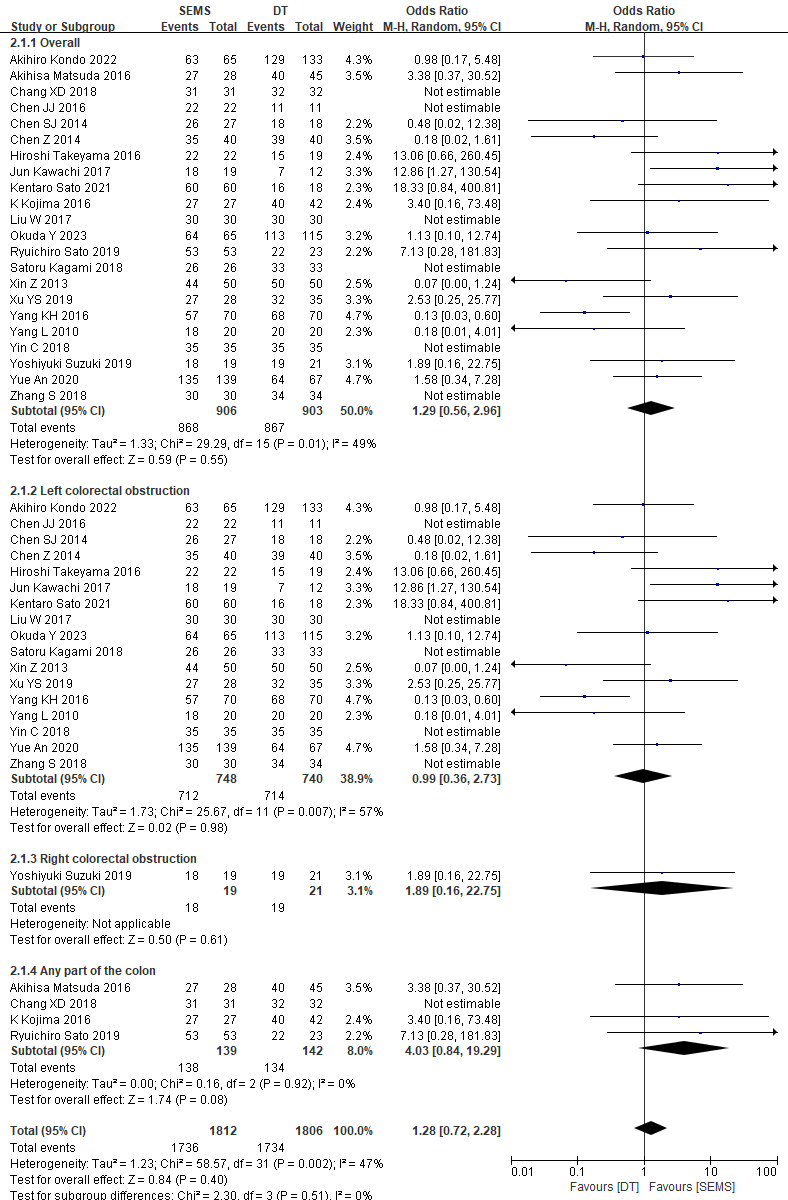


**Figure 1.** Forest plot of meta-analysis results regarding technical success in SEMS and DT groups. SEMS, self-expanding metal stent; DT, decompression tube; CI, confidence interval; M-H, Mantel-Haenszel; df, degree of freedom


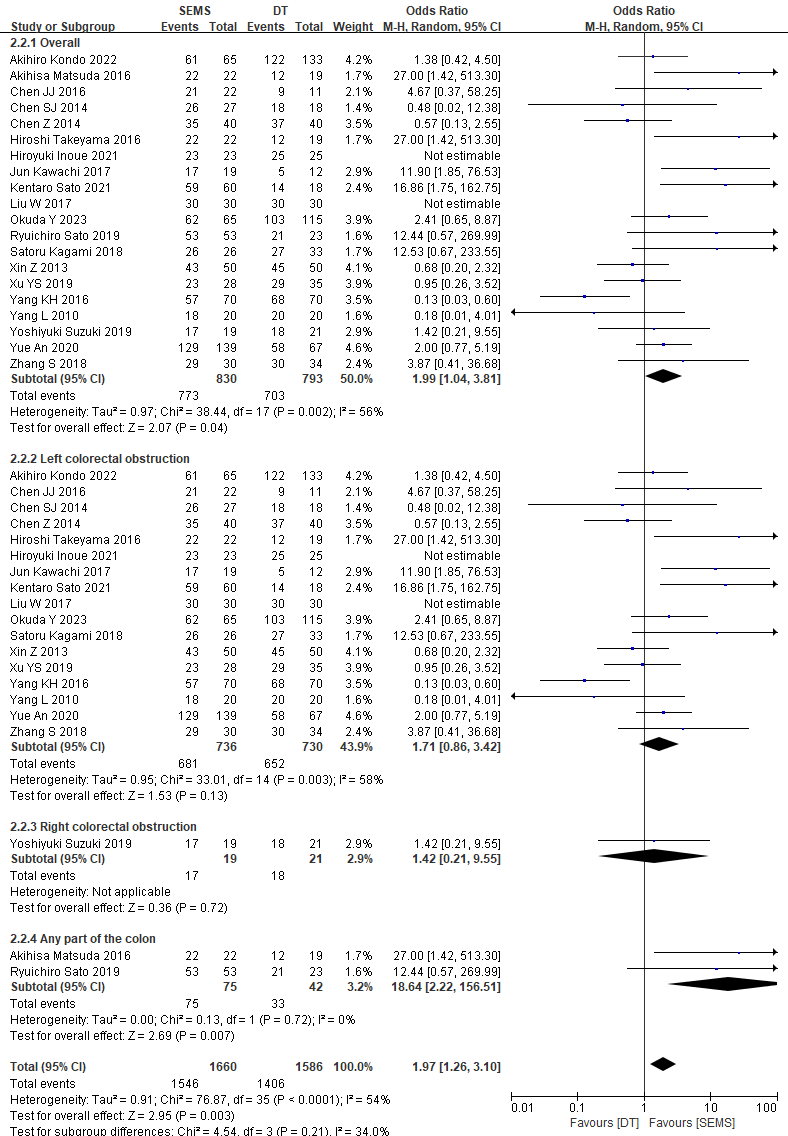


**Figure 2.** Forest plot of meta-analysis results regarding clinical success in SEMS and DT groups. SEMS, self-expanding metal stent; DT, decompression tube; CI, confidence interval; M-H, Mantel-Haenszel; df, degree of freedom


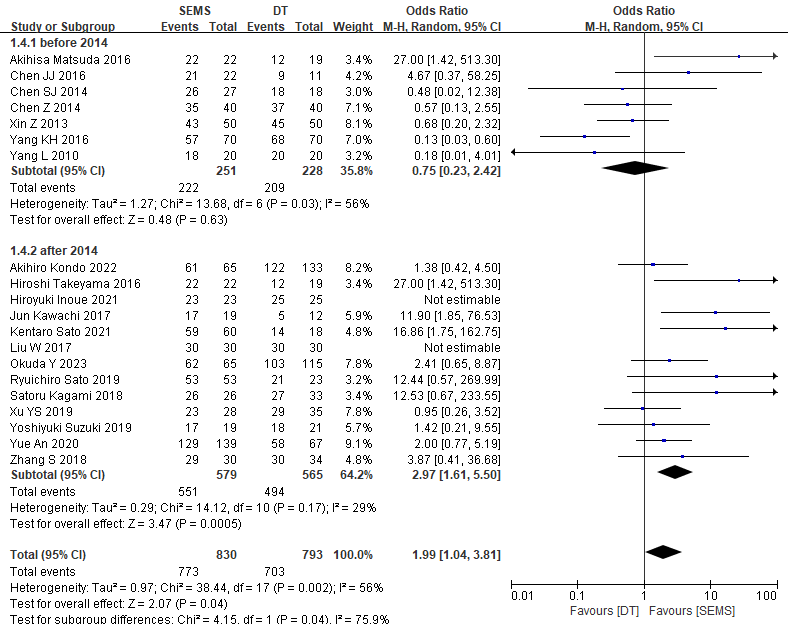


**Figure 3.** Forest plot of meta-analysis results regarding clinical success in SEMS and DT groups. SEMS, self-expanding metal stent; DT, decompression tube; CI, confidence interval; M-H, Mantel-Haenszel; df, degree of freedom


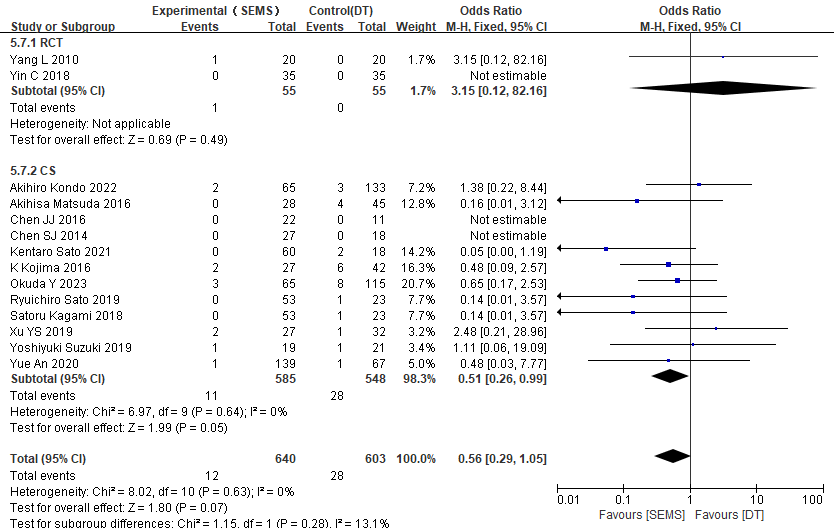


**Figure 4.** Forest plot of meta-analysis results regarding operation-related perforation in SEMS and DT groups. SEMS, self-expanding metal stent; DT, decompression tube; CI, confidence interval; M-H, Mantel-Haenszel; df, degree of freedom


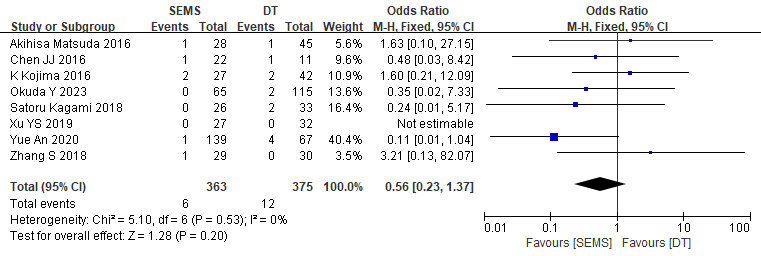


**Figure 5.** Forest plot of meta-analysis results regarding device migration in SEMS and DT groups. SEMS, self-expanding metal stent; DT, decompression tube; CI, confidence interval; M-H, Mantel-Haenszel; df, degree of freedom


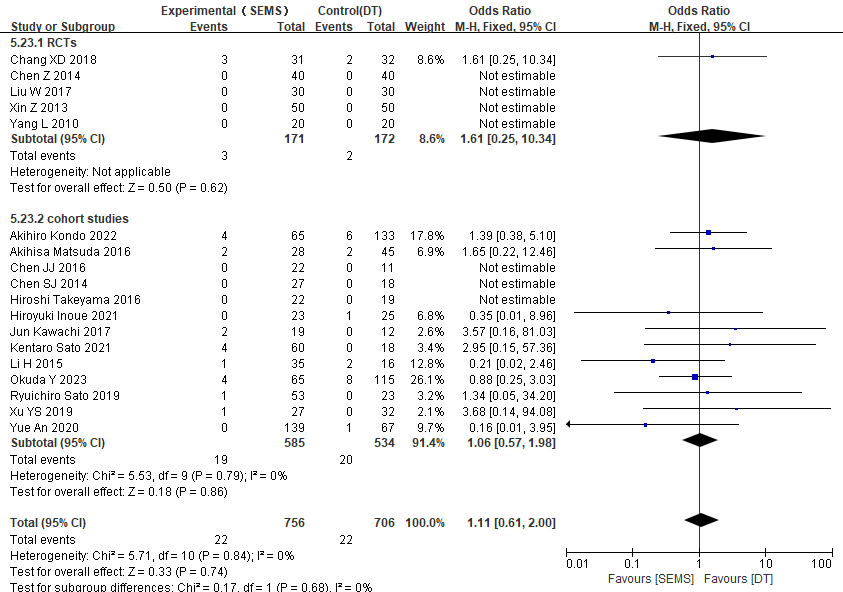


**Figure 6.** Forest plot of meta-analysis results regarding postoperative anastomotic leakage in SEMS and DT groups. SEMS, self-expanding metal stent; DT, decompression tube; CI, confidence interval; M-H, Mantel-Haenszel; df, degree of freedom


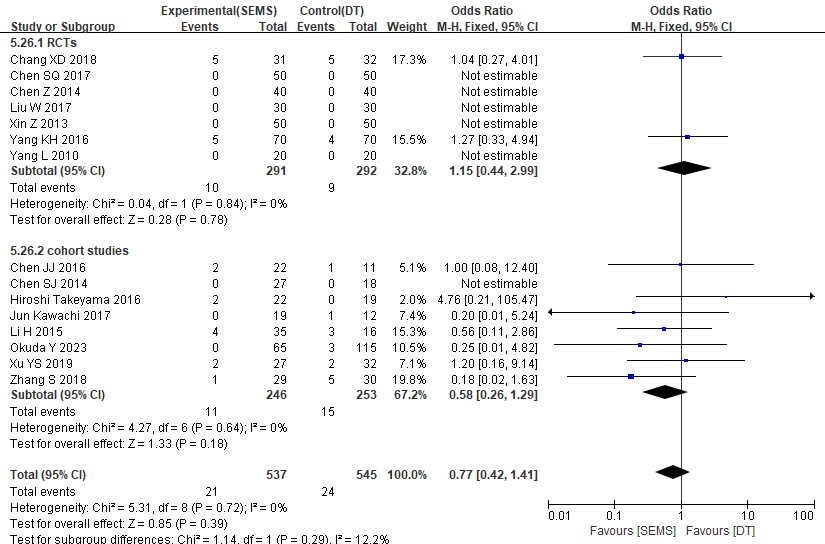


**Figure 7.** Forest plot of meta-analysis results regarding postoperative infection occurrence in SEMS and DT groups. SEMS, self-expanding metal stent; DT, decompression tube; CI, confidence interval; M-H, Mantel-Haenszel; df, degree of freedom


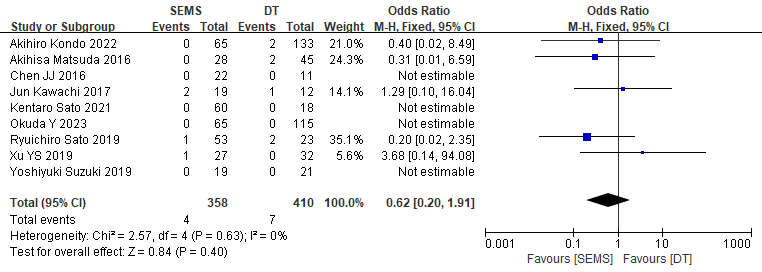


**Figure 8.** Forest plot of meta-analysis results regarding postoperative 30-day mortality in SEMS and DT groups. SEMS, self-expanding metal stent; DT, decompression tube; CI, confidence interval


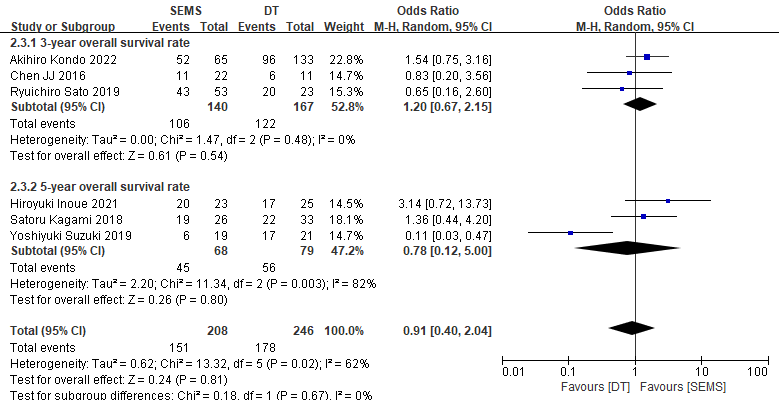


**Figure 9.** Forest plot of meta-analysis results regarding overall survival rates in SEMS and DT groups. SEMS, self-expanding metal stent; DT, decompression tube; CI, confidence interval; M-H, Mantel-Haenszel; df, degree of freedom


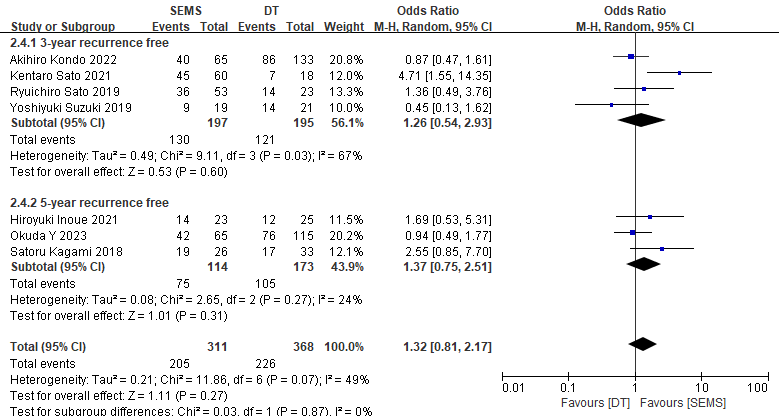


**Figure 10.** Forest plot of meta-analysis results regarding recurrence-free rates in SEMS and DT groups. SEMS, self-expanding metal stent; DT, decompression tube; CI, confidence interval; M-H, Mantel-Haenszel; df, degree of freedom


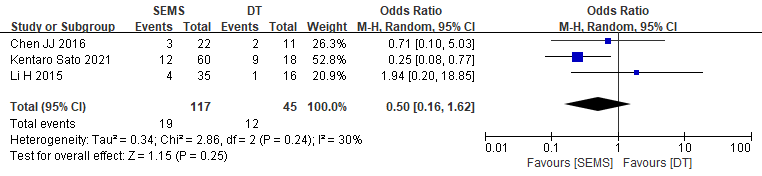


**Figure 11.** Forest plot of meta-analysis results regarding tumor metastasis in SEMS and DT groups. SEMS, self-expanding metal stent; DT, decompression tube; CI, confidence interval; M-H, Mantel-Haenszel; df, degree of freedom


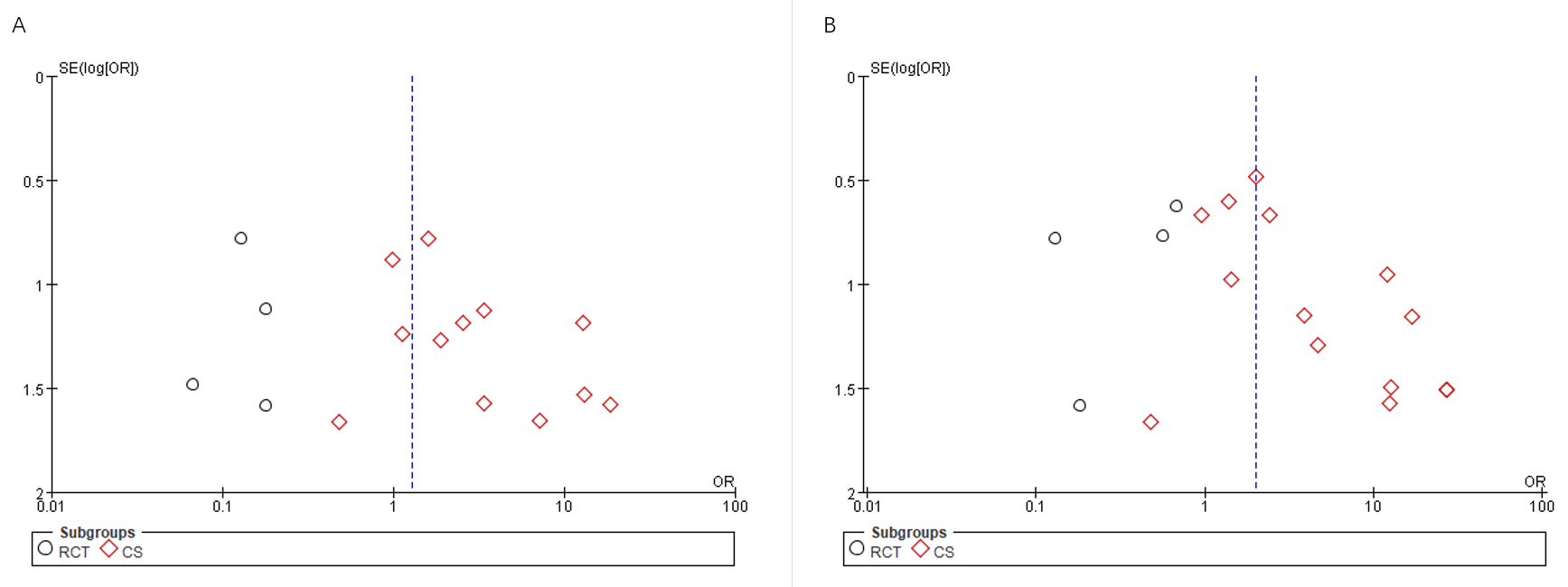


**Figure 12.** Funnel plot of the included randomized controlled trials and cohort studies. A: technical success; B: clinic success. RR, risk ratio; SE, standard error; OR, odds ratio
