# Supplementary material for: Self-expanding metal stents versus decompression tubes as a bridge to surgery for patients with obstruction caused by colorectal cancer: a systematic review and meta-analysis
Source: World J Emerg Surg. 2023 Sep 27;18:46. doi: 10.1186/s13017-023-00515-6 (PMC10536785; doi:10.1186/s13017-023-00515-6)
Supplement: Supplementary file 5 [file 13017_2023_515_MOESM3_ESM.docx]

Study ID	D1	D2	D3	D4	D5	D6	D7	Overall
Akihiro Kondo, 2022	!	+	+	+	+	+	+	!
Akihisa Matsuda, 2016	+	!	+	+	+	+	+	!
Chen JJ, 2016	+	+	+	+	+	+	+	+
Chen SJ, 2014	!	+	+	+	+	+	+	!
Hiroshi Takeyama, 2016	+	!	+	+	+	+	+	!
Hiroyuki Inoue, 2021	!	-	+	+	+	+	+	-
Jun Kawachi, 2017	+	+	+	+	+	+	+	+
K. Kojima, 2016	!	-	+	+	+	+	+	-
Kentaro Sato, 2021	!	-	+	+	+	+	+	-
Li H,2015	+	+	+	+	+	+	+	+
Ryuichiro Sato, 2019	!	+	+	+	+	+	+	!
Satoru Kagami, 2018	!	-	+	+	+	+	+	-
Xu YS, 2019	!	+	+	+	+	+	+	!
Okuda Y, 2023	!	+	+	+	+	+	+	!
Yoshiyuki Suzuki, 2019	+	!	+	+	+	+	+	!
Yue An, 2020	+	+	+	+	+	+	+	+
Zhang S, 2018	+	+	+	+	+	+	+	+
Zhang S, 2021	+	+	+	+	+	+	+	+

Domains:
D1: Bias due to confounding.
D2: Bias due to selection of participants.
D3: Bias in classification of interventions.
D4: Bias due to deviations from intended interventions.
D5: Bias due to missing data.
D6: Bias in measurement of outcomes.
D7: Bias in selection of the reported result.

Low +
Moderate !
Serious -
